# Deoxycholic acid impairs mitochondrial fitness and suppresses mitophagy through Parkin ubiquitination driving CD8^+^ T cell Exhaustion in Colorectal Cancer

**DOI:** 10.1016/j.redox.2026.104322

**Published:** 2026-07-25

**Authors:** Yang Gao, Jiaxin Wang, Shihui Lai, Mingwei Li, Jiaqi Shi, Xianjun Li, Ying Guo, Sainan Ma, Xinxin Wang, Shule Zhang, Tianyu He, Yu Hong, Shuang Li, Caiqi Liu, Lijuan Cao, Haiping Hao, Chujie Ding

**Affiliations:** a Heilongjiang Province Key Laboratory of Molecular Oncology, Harbin, Heilongjiang, China; b Department of Gastrointestinal Medical Oncology, Harbin Medical University Cancer Hospital, Harbin, China; c Department of Hepatobiliary and Pancreatic Surgery, Harbin Medical University Cancer Hospital, Harbin, China; d Department of Phase I Trials Center, Harbin Medical University Cancer Hospital, Harbin, China; e Department of Breast Surgery, Harbin Medical University Cancer Hospital, Harbin, China; f Heilongjiang Cancer Institute, Harbin, China; g Imaging Department, Harbin Medical University Cancer Hospital, Harbin, China; h State Key Laboratory of Natural Medicines, China Pharmaceutical University, Nanjing, China; i Jiangsu Provincial Key Laboratory of Targetome and Innovative Drugs, Institute of Innovative Drug, China Pharmaceutical University, Nanjing, China; j Laboratory of Metabolic Regulation and Drug Target Discovery, School of Pharmacy, China Pharmaceutical University, Nanjing, China; k Scientific Research Center, Harbin Medical University Cancer Hospital, Harbin, China

**Keywords:** Deoxycholic acid, CD8^+^ T cell terminal exhaustion, Mitochondrial dysfunction, Mitophagy, Anti-PD-1 therapy, Colorectal cancer

## Abstract

Colorectal cancer (CRC) exhibits significant heterogeneity in response to immunotherapy that cannot be fully explained by microsatellite status alone. Although elevated bile acid levels are recognized as an important risk factor for CRC, their impact on immunotherapy responsiveness remains poorly understood. Here, we demonstrate that high bile acid levels profoundly impair anti-PD-1 efficacy in both CRC patient cohort and mouse models, accompanied by reduced infiltration and functional impairment of tumor-infiltrating CD8^+^ T cells. Bile acid profiling identified deoxycholic acid (DCA) as the key bile acid species mediating this immunosuppressive effect. *In vitro* and *in vivo* studies have shown that DCA not only suppressed CD8^+^ T cell effector function but also drove them toward terminal exhaustion, thereby limiting responsiveness to anti-PD-1. Mechanistically, DCA disrupted mitochondrial fitness in CD8^+^ T cells by suppressing oxidative phosphorylation and inducing excessive mitochondrial reactive oxygen species (mtROS) production. In parallel, DCA enhanced ubiquitination-dependent degradation of Parkin, thereby inhibiting mitophagy and causing the accumulation of damaged mitochondria. These convergent defects in mitochondrial homeostasis ultimately promoted CD8^+^ T cell dysfunction and terminal exhaustion. Notably, pharmacological reactivation of mitophagy via Urolithin A reversed these defects and restored the antitumor efficacy of anti-PD-1 *in vivo*. Collectively, our findings identified a DCA-Parkin-mitophagy axis that drives CD8^+^ T cell terminal exhaustion and compromises immunotherapy efficacy, providing a potential metabolic intervention strategy to improve immunotherapy responses in CRC patients with elevated bile acid levels.

## Highlights

• DCA promotes terminal exhaustion of CD8^+^ T cells in CRC.
• DCA impairs mitophagy and mitochondrial fitness in tumor-infiltrating CD8^+^ T cells.
• DCA triggers Parkin ubiquitination and degradation to suppress mitophagy flux.
• Urolithin A restores mitochondrial fitness to rejuvenate CD8^+^ T cell function.
• Targeting the DCA-Parkin-mitophagy axis enhances anti-PD-1 efficacy in CRC.

## Graphical abstract

**Figure:**
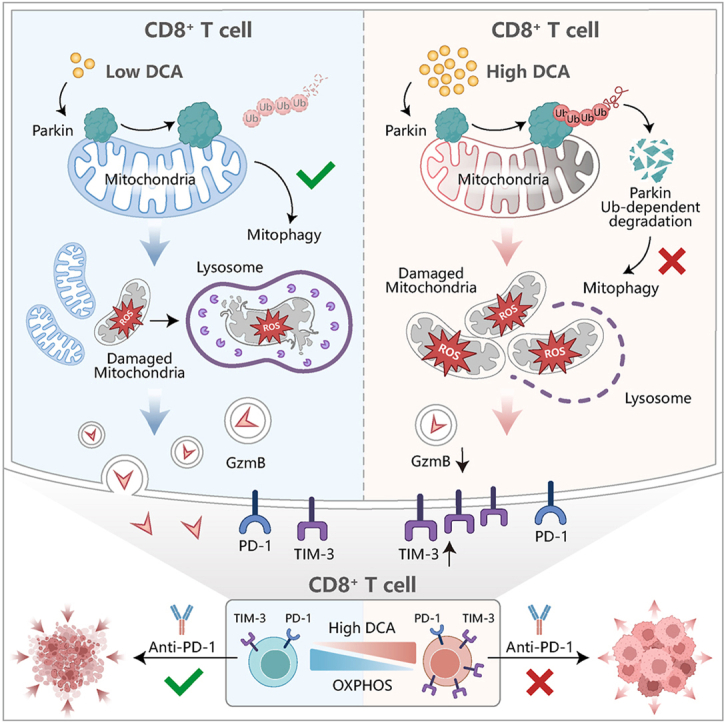

## 1 Introduction

Colorectal cancer (CRC) is one of the common digestive tract malignant tumors with increasing incidence and mortality worldwide [1]. In recent years, immune checkpoint blockade (ICB), particularly anti-PD-1 therapy, has conferred substantial clinical benefits to a subset of CRC patients [2]. Although microsatellite instability-high (MSI-H) status has been established as a robust predictive biomarker for anti-PD-1 responsiveness, clinical benefits is not universal among MSI-H tumors [3]. Conversely, pathological remission also has been observed in a subset of microsatellite stable (MSS) tumors [4,5]. These phenomena underscore the existence of underappreciated regulatory factors beyond intrinsic tumor genetics that dictate the heterogeneity of therapeutic responses to anti-PD-1 therapy in CRC.

The efficacy of tumor immunotherapy is governed not only by intrinsic genomic features of malignant cells, but also by the intricate tumor metabolic microenvironment [6,7]. Bile acids (BAs), as major metabolites derived from the gut microbiota, function far beyond their canonical role in lipid digestion, acting as pivotal signaling molecules that modulate tumor progression and orchestrate systemic immune responses [[8], [9], [10], [11]]. Previous studies have demonstrated that elevated conjugated primary BAs are closely associated with liver metastasis of CRC [12]. Similarly, the aberrant accumulation of primary and secondary BAs constitutes a hallmark metabolic feature of hepatocellular carcinoma [13], implicating BA dysregulation as a critical driver in tumorigenesis of digestive system malignancies. In the context of immune regulation, our prior work revealed that deoxycholic acid (DCA) directly modulates T cell activation, suggesting a potential role for BAs in bridging host metabolism and adaptive immunity [14]. While subsequent study has further corroborated that DCA regulates the antitumor effector functions of CD8^+^ T cells, reinforcing the significance of BAs in tumor immunology [15]. Despite these advances, whether bile acid signaling serves as a primary determinant of immunotherapy responsiveness in CRC remains poorly understood. Given that the efficacy of anti-PD-1 therapy is fundamentally limited by CD8^+^ T cell terminal exhaustion, a state characterized by compromised mitochondrial fitness and the aberrant accumulation of damaged mitochondria [[16], [17], [18], [19]], it is necessary to elucidate how bile acid signals interface with CD8^+^ T cell terminal exhaustion state. Deciphering how the high bile acid environment compromises this metabolic-immune intersection is essential for developing targeted strategies to overcome immunotherapy resistance in colorectal cancer.

In this study, we investigated the impact of elevated bile acid levels on anti-PD-1 efficacy in CRC and elucidated the underlying mechanisms governing CD8^+^ T cell mitochondrial homeostasis. We identified deoxycholic acid (DCA) as a pivotal metabolite that impairs anti-PD-1 efficacy by promoting terminal exhaustion of CD8^+^ T cells. Mechanistically, DCA triggered mitochondrial damage and inhibited Parkin-dependent mitophagy, resulting in the accumulation of dysfunctional mitochondria and subsequent CD8^+^ T cell dysfunction. Notably, pharmacological reactivation of mitophagy restored mitochondrial fitness and improved responsiveness to anti-PD-1 therapy. Together, these findings reveal a previously unappreciated role of bile acid metabolic dysregulation in driving CD8^+^ T cell terminal exhaustion and immunotherapy resistance, and highlight metabolic intervention as a potential strategy to restore antitumor immunity in CRC.

## 2 Materials and methods

### 2.1 Patients and tissue samples

A total of 48 patients with pathologically confirmed colorectal cancer (Harbin Medical University Cancer Hospital) provided pre-treatment serum total bile acid levels and paired pre-/post-treatment radiological imaging data for the evaluation of immunotherapy efficacy according to RECIST version 1.1. Clinical information for the above samples is provided in Supplementary Table S1. Besides, 17 primary colorectal carcinoma tissues analyzed in this study were procured from standard surgical resections performed at Harbin Medical University Cancer Hospital, along with serum bile acid levels collected at patient admission. Clinical information for the above samples is provided in Supplementary Table S2. The study protocol received ethical approval from the Institutional Review Board of Harbin Medical University Cancer Hospital, complied with the principles outlined in the Declaration of Helsinki, and obtained comprehensive written informed consent from all participants prior to tissue collection.

### 2.2 Cell lines and mouse

MC38, MC38-GFP and MC38-Luc cell lines were acquired from the National Collection of Authenticated Cell Cultures at the Chinese Academy of Sciences (Shanghai, China). MC38 cells were cultured in DMEM medium (Gibco, C11995500BT), supplemented with 10% fetal bovine serum (ExCell Bio, FSP500) and 1% penicillin-streptomycin (NCM Biotech, C100C5). All cells were incubated at 37 °C in a humidified incubator with 5% CO_2_ and 90% relative humidity.

Six-week-old male C57BL/6 mice were purchased from Liaoning Changsheng Biotech. Following a 7-day acclimatization period under standard housing conditions (22 °C, 12 h light/dark cycle), all animal experiments were conducted in compliance with protocols approved by the Institutional Animal Care and Use Committee.

### 2.3 Mouse CD8^+^ T cells isolation, culture and administration

Splenic single-cell suspensions from C57BL/6 mice were subjected to erythrocyte lysis (Miltenyi Biotec, 130-094-183) and CD8^+^ T cell negative selection using a commercial kit (Stemcell, 19853). Purified cells were activated for 3 days in complete RPMI 1640 medium containing 10 μg/mL anti-CD3 (BioLegend, 100340), 2 μg/mL anti-CD28 (BioLegend, 102116), and 20 ng/mL IL-2 (PeproTech, 212-12), followed by expansion in IL-2-supplemented medium for 7-14 days for subsequent experiments.

*In vitro* administration of CD8**^+^** T cells was performed as follows: DCA (Sigma-Aldrich, D2510) was administered at final concentrations of 25 and 50 μM for 24 h; chenodeoxycholic acid (CDCA) (solarbio, IC0300) and ursodeoxycholic acid (UDCA) (solarbio, IU0020) were administered at concentrations of 25 μM for 24 h; Urolithin A (MCE, HY-100599) was administered at 50 μM for 24 h; CHX (MCE, HY-12320) was administered at 50 μg/mL with varied treatment durations; Bafilomycin A1 (MCE, HY-100558) was administered at 100 nM for 4 h; Dynasore (MCE, HY-15304) was administered at 80 μM for 30 min prior to the start of DCA administration and was maintained for 24 h; MG132 (MCE, HY-13259) was administered at 10 μM for 6 h.

### 2.4 Mouse models and treatments

For establishment of the subcutaneous tumor model, MC38 or MC38-Luc cells (2.5 × 10^5^ per mouse) were suspended in 100 μL PBS and inoculated into the right flank of 6-week-old C57BL/6 mice. Subcutaneous tumors typically reached a volume of 100-150 mm^3^ approximately 7 days post-inoculation.

To generate the orthotopic colorectal tumor model, donor C57BL/6 mice bearing MC38 subcutaneous tumors were euthanized by cervical dislocation 14 days after inoculation. Subcutaneous tumors were aseptically excised and minced into approximately 1 mm^3^ fragments. Recipient mice were anesthetized and secured, followed by a 1 cm longitudinal incision made 1 cm left of the midline. The serosal layer at the cecocolonic junction was gently scratched using a sterile 1 mL syringe needle. A single tumor fragment was implanted onto the scratched site and secured with 1-2 drops of a surgical adhesive (Jitian Bio, JT0701). After the adhesive solidified, the cecum and colon were carefully repositioned into the abdominal cavity. The peritoneal cavity was irrigated with antibiotic-containing saline prior to wound closure.

For *in vivo* CD8^+^ T cell depletion, mice received intraperitoneal injections of InVivoMab anti-mouse CD8α antibody (STARTER, S0B0660) (100 μg per mouse), while control mice were administered InVivoMab rat IgG2b isotype control (BioXcell, BE0089) (100 μg per mouse) twice weekly.

For anti-PD-1 immunotherapy and isotype control administration, mice were treated intraperitoneally with anti-PD-1 antibody (BioXcell, BE0146) or Rat IgG2α isotype control antibody (BioXcell, BE0089) (100 μg per mouse) twice per week.

For Urolithin A treatment, UA (Sigma-Aldrich, SML1791) (400 μg per mouse) was administered daily via oral gavage for five consecutive days per week.

In dietary intervention groups, mice were fed either a 0.5% CA (Sigma-Aldrich, C1129) or 0.5% DCA (Sigma-Aldrich, D2510) supplemented diet for 7 days prior to tumor implantation and maintained on the respective diet throughout the experimental period.

Tumor volumes were calculated using the formula: Volume = (length × width^2^)/2.

### 2.5 *In vivo* imaging for monitoring tumor growth

Bioluminescence imaging and quantification tumor burden was assessed longitudinally on days 7, 14, and 21 post-implantations using the IVIS Spectrum System. Mice received an intraperitoneal injection of 150 mg/kg D-luciferin (KKL Med Inc, KM8318) (suspended in PBS) and were anesthetized with isoflurane (RWD, R510-22-10) 5 min prior to acquisition. Images were captured using auto-exposure settings. Bioluminescent signals were quantified using Living Image software (v4.4) by calculating the average radiance efficiency within ROIs encompassing the tumor site.

### 2.6 Isolation of tumor-infiltrating immune cell suspension

Tumors were minced and digested with collagenase IV (Solarbio, C8160) (1 mg/mL),hyaluronidase (Solarbio, H8030) (100 μg/mL) and DNase I (Solarbio, D8071) (100 μg/mL) in serum-free RPMI-1640 (1 h, 37 °C). Following filtration (70 μm), leukocytes were enriched via a discontinuous 40%/70% Percoll gradient (800 g, 30 min). CD8^+^ T cells were purified from the interphase fraction by negative selection (Miltenyi Biotec, 130-094-183) and activated at 1 × 10^6^ cells/mL with plate-bound anti-CD3 (10 μg/mL) and soluble anti-CD28 (2 μg/mL).

### 2.7 Flow cytometric analysis

For flow cytometric analysis, single-cell suspensions isolated from tumor tissues were adjusted to a density of 2 × 10^7^ cells/mL. An aliquot of 100 μL cell suspension (containing 2 × 10^6^ cells) was used for staining. To characterize the immune cells, these were stained with fluorochrome-conjugated antibodies (detailed in Supplementary Table S3). All antibody staining reactions were performed in the dark for 20 mi at 4 °C. Subsequently, cells were washed once with PBS, centrifuged at 600 × g for 5 min at 4 °C, and resuspended in 500 μL PBS. Samples were acquired on a flow cytometer (BD FACSMelody™), and data were analyzed using FlowJo software (version 10).

### 2.8 Enzyme-linked immunosorbent assay (ELISA)

The concentrations of TNF-α (Excellbio, EM008-96), IL-2 (Excellbio, EM002-96), and Granzyme B (Liankebio, EK2173) in cell culture supernatants were quantified using specific ELISA kits following standard protocols. Briefly, standards and samples were added to the assay plates, followed by sequential incubation with biotinylated detection antibodies and enzyme conjugates. After washing, substrate solution was added for color development, and the reaction was stopped with stop solution. Absorbance was measured at 450 nm, and cytokine concentrations were determined from the standard curves.

### 2.9 Immunohistochemistry staining

Paraffin-embedded tissue sections were processed for antigen retrieval (citrate buffer, pH 6.0), permeabilized with 0.1% Triton X-100, and blocked with 5% BSA. Sections were probed overnight at 4 °C with primary antibodies: anti-Ki67 (Abcam, ab15580), anti-CD8 (CST, 70306S), anti-Tim3 (Proteintech, 11872-1-AP), anti-PD-1 Abcam, ab214421), anti-Galectin-9 (Abcam, ab69630), anti-BTLA (Abcam, ab216505), anti-TNFRSF14 (Abcam, ab47677), and anti-PD-L1 (Proteintech, 28076-1-AP). Immunoreactivity was visualized via HRP-conjugated secondaries and DAB, with hematoxylin counterstaining. Slides were scanned using a Digital Slide Scanner (Motic). Positive staining was quantified using ImageJ software.

### 2.10 Serum total bile acid measurement

Peripheral blood was collected retro-orbitally, allowed to clot at room temperature, and centrifuged at 6000 × g for 10 min. Isolated serum was analyzed immediately or stored at −80 °C. TBA levels were quantified using an enzymatic cycling assay based on NAD reduction (Njjcbio, E003-2-1). Briefly, serum samples or standards were incubated with reaction buffer at 37 °C for 30 min. Following the addition of chromogenic reagent, the change in absorbance at 405 nm was monitored between 1 and 3 min. Concentrations were calculated using a quadratic standard curve derived from mixed bile acid standards.

### 2.11 Targeted bile acid metabolomics

Plasma bile acids were extracted with methanol containing internal standards. Analytes were separated on an ACQUITY UPLC HSS T3 column and detected using a SCIEX QTRAP® 6500+ system operating in negative MRM mode. Data were acquired and quantified using Analyst® and MultiQuant™ software, respectively.

### 2.12 Intracellular DCA measurement

Cells were washed three times with ice-cold PBS to remove extracellular DCA and collected by centrifugation. Cell pellets were extracted with 300 μL methanol by ultrasonication for 30 min, followed by centrifugation, and the supernatants were subjected to HPLC analysis. DCA was quantified using an Agilent 1260 HPLC system equipped with an evaporative light-scattering detector (ELSD) and a C18 column (250 × 4.6 mm, 5 μm). The mobile phase consisted of acetonitrile and 0.1% formic acid in water (60:40, v/v) at a flow rate of 1.0 mL/min. DCA concentrations were calculated from an external standard curve.

### 2.13 Co-culture of CD8^+^ T cells with MC38 cells

To assess metabolic alterations, MC38-GFP cells freshly sorted from tumor tissues (1 × 10^5^ cells/well) were seeded in 6-well plates and cultured for 24 h. Lymph nodes derived CD8^+^ T cells, pre-conditioned with DCA or vehicle for 24 h, were subsequently co-cultured with MC38-GFP cells at an effector-to-target (E: T) ratio of 10:1. Following a 48 h incubation, both cell populations were harvested for further testing.

### 2.14 Transfection of CD8^+^ T cells

For Parkin overexpression, CD8^+^ T cells were seeded in six-well plates at a density of 1 × 10^6^ cells per well. Transient transfection was performed using the jetPEI reagent (Polyplus, 101000053) following the manufacturer's protocol. Briefly, 8 μg of plasmid DNA and 16 μL of jetPEI reagent were diluted separately in 100 μL of 150 mM NaCl. The solutions were mixed and incubated at room temperature for 30 min to facilitate complex formation. The resulting DNA-jetPEI complexes were added dropwise to the culture medium, and cells were analyzed 48 h post-transfection.

For knockdown of canonical bile acid-related cell-surface receptors, CD8^+^ T cells were transfected with siRNA targeting TGR5 and S1PR2 (Sangon Biotech) using ProteanFect Max transfection reagent (Nanoportal Biotech) according to the manufacturer's instructions. After transduced or transfection for 48 h, cells were collected for further experimental validation.

### 2.15 Total RNA extraction and RT-qPCR

Total RNA was extracted from T cells using TRIzol reagent (Thermo Fisher Scientific, 15596026CN). Subsequently, 500 ng of total RNA was reverse-transcribed into cDNA following the manufacturer's protocol of the reverse transcription kit. Using the synthesized cDNA as template, amplification was performed with SYBR Green I Master Mix (Roche, 4913850001) on a CFX96 real-time PCR system (Bio-Rad). The relative mRNA expression levels of target genes were calculated using the 2^−ΔΔCt^ method. The sequences of all primers used in this study are listed in Supplementary Table S4.

### 2.16 CD8^+^ T cells chronic exhaustion models

To induce chronic exhaustion, primary spleen derived CD8^+^ T cells (1 × 10^6^ cells/mL) were initially stimulated with plate-bound anti-CD3 (10 μg/mL) and soluble anti-CD28 (2 μg/mL) for 2 days. Then chronic stimulation was maintained by replacing the medium with plate-coated anti-CD3 (10 μg/mL) every 48 h for 8 days.

### 2.17 Seahorse XF assay

Cellular metabolism was analyzed using the Seahorse XFe96 Analyzer (Agilent). Sensor cartridges were hydrated overnight in XF calibration solution at 37 °C without CO_2_. CD8^+^ T cells or MC38 cells were resuspended in Seahorse XF Mito Stress Test medium (2 mM glutamine, 1 mM sodium pyruvate, 10 mM glucose) or XF Glycolysis Stress Test medium (2 mM glutamine). Cells (1 × 10^5^ per well) were plated in pre-coated XF96 plates, centrifuged at 200 × g for 1 min, and equilibrated for 30 min at 37 °C. For mitochondrial stress tests, oligomycin (1 μM), FCCP (0.25 μM), and antimycin A/rotenone (0.5 mM each) were sequentially injected to measure OCR. For glycolysis stress tests, glucose (10 mM), oligomycin (1 μM), and 2-deoxy-d-glucose (50 mM) were sequentially injected to measure ECAR. Data were normalized to cell number.

### 2.18 Single-cell suspensions preparation and single-cell RNA sequencing

CD8^+^ T cells isolated from tumor tissue were loaded according to the 10x Genomics at a density of 18000 cells per chip. Using the Chromium Next GEM Single Cell 5’ Reagent Kits v2, approximately 10000 single cells were encapsulated into oil-water GEMs via microfluidics, completing single-cell isolation, reverse transcription, and barcoding to construct single-cell RNA sequencing libraries. Following library quantification with Qubit and quality assessment with Agilent 2100, paired-end 150 bp sequencing was performed on the Illumina NovaSeq 6000 platform, with an average sequencing depth of no less than 50,000 reads per cell. Raw sequencing data were processed using Cell Ranger (v7.1.0) to generate the gene expression matrix. High-quality cells after quality control were subjected to normalization, principal component analysis, and clustering via Seurat (v4.3.0). Significantly differentially expressed genes were subsequently analyzed for Gene Ontology enrichment (using clusterProfiler v4.6.2) and gene set enrichment analysis based on specific gene signature scores (using GSEA v4.3.2) to elucidate their biological functions and associated pathway alterations.

### 2.19 Bulk RNA-Seq data analysis

Total RNA of tumor-infiltrating CD8^+^ T cells was extracted using the TRIzol. RNA concentration and purity were measured using a Nanodrop spectrophotometer (A260/280 and A260/230 ratios), and RNA integrity was assessed with an Agilent 2100 Bioanalyzer (RIN >7.0). RNA samples meeting the quality criteria were used for subsequent library construction. Sequencing libraries were prepared using the NEBNext® Ultra™ RNA Library Prep Kit for Illumina, with the main steps including mRNA enrichment, fragmentation, double-stranded cDNA synthesis, end repair, adapter ligation, and PCR amplification. The quality of the constructed libraries was verified using an Agilent 2100 Bioanalyzer. Qualified libraries were sequenced on an Illumina NovaSeq 6000 platform to generate 150-bp paired-end reads, with a minimum sequencing depth of 6 Gb per sample.

### 2.20 Assessment of mitochondrial function

Isolated CD8^+^ T cells were resuspended at a density of 1 × 10^6^ cells/mL in prewarmed (37 °C) serum-free RPMI-1640 medium. Mitochondrial status was assessed by flow cytometry following staining with MitoTracker Deep Red FM (Beyotime, C1032), MitoTracker Green (Beyotime, C1048), and MitoSOX Red (Thermo Fisher, M36008). Data were analyzed using FlowJo software.

### 2.21 Transmission electron microscopy

CD8^+^ T cells were fixed in 2.5% glutaraldehyde (25% glutaraldehyde ampules, Ted Pella Co. USA) for 12 h at 4 °C. Samples were washed three times with ice-cold 0.1 M phosphate buffer (pH 7.3) and post-fixed in 1% osmium tetroxide for 1 h. Then samples were infiltrated sequentially with ethanol/Spurr resin mixtures (1:1 and 1:3, v/v) for 30 min each, followed by infiltration with 100% Spurr resin for 3 h. Samples were then embedded and polymerized in Spurr resin at 60 °C for 24 h. Ultrathin sections were mounted on nickel grids and stained with 2% uranyl acetate for 10 min and Reynolds’ lead citrate for 5 min. Sections were examined using a JEM-1400 Plus transmission electron microscope.

### 2.22 Western blot analysis

Whole cell protein was extracted with cell lysis RIPA buffer (Beyotime, P0013B) containing protease and phosphatase inhibitors. Proteins were quantified using a BCA kit (Beyotime, P0009), separated by SDS-PAGE, transferred to PVDF membranes (ZY101123, Millipore), blocked with 5% BSA, and then incubated with primary antibodies overnight at 4 °C. After incubation with fluorescence dye-conjugated secondary antibodies, signals were captured using a ChemiDoc MP imaging system (Bio-Rad). Primary antibodies were used: anti-VDAC1 (Proteintech, 55259-1-AP), anti-PINK1 (Proteintech, 23274-1-AP), anti-Parkin (Proteintech, 14060-1-AP), anti-LC3B (Proteintech, 18725-1-AP), anti-Tom20 (Abcam, ab186735), anti-β-actin (Proteintech, 66009-1-Ig), anti-ubiquitin (Proteintech, 10201-2-AP), anti-K63-linkage ubiquitin (Cell Signaling Technology, 5621), anti-K48-linkage ubiquitin (Cell Signaling Technology, 8081), anti-TGR5 (Affinity, DF14067), anti-S1PR2 (Affinity, DF4921).

### 2.23 Immunoprecipitation assays

CD8^+^ T cells were lysed in NP-40 lysis buffer supplemented with protease and phosphatase inhibitor cocktails. Cell lysis was performed on ice with sonication, followed by centrifugation at 13000 × g at 4 °C to obtain total protein extracts. To minimize nonspecific binding, equal amounts of protein extracts were pre-cleared with bovine serum albumin–blocked Protein A/G agarose beads at 4 °C. Protein A/G agarose beads were pre-incubated with an anti-Parkin antibody or an isotype-matched control antibody for 12h at 4 °C, and subsequently incubated with the pre-cleared protein extracts for 6h at 4 °C with rotation to allow immune complex formation. After immunoprecipitation, immune complexes were collected by centrifugation, washed with lysis buffer containing 1% NP-40, and resuspended in SDS sample buffer. Samples were heated to denature proteins, centrifuged, and the resulting supernatants were subjected to Western blot analysis.

### 2.24 Immunofluorescence

Cells were seeded on glass coverslips placed in 6-well plates. After washing with PBS, cells were fixed with 4% paraformaldehyde for 15 min at room temperature, permeabilized with 0.1% Triton X-100 for 15 min, and blocked with 5% bovine serum albumin for 1 h at room temperature. The samples were then incubated overnight at 4 °C with the following primary antibodies: anti-LAMP1 mouse monoclonal antibody (Abcam, ab25630) and anti-Tom20 rabbit polyclonal antibody (Abcam, ab186735). After washing, the coverslips were incubated with corresponding fluorescent secondary antibodies for 1 h at room temperature in the dark, including DyLight 594-conjugated goat anti-mouse IgG (Abbkine, A23410) and DyLight 488-conjugated goat anti-rabbit IgG (Abbkine, A23220). Nuclei were counterstained with DAPI. Images were acquired using a laser-scanning confocal microscope (Zeiss).

### 2.25 Statistics analysis

Statistical analyses were performed using GraphPad Prism 10. Data are presented as mean ± SEM. Student' s *t*-test was used for comparisons between two groups. For comparisons among multiple groups, one-way ANOVA was applied. Pearson correlation analysis was performed to assess linear associations; r and p represent the Pearson correlation coefficient and the two-tailed P value, respectively. For comparisons of treatment response between groups in the cohort of 48 patients with colorectal cancer, the Wilcoxon rank-sum test was used. *P* < 0.05 was considered statistically significant.

## 3 Results

### 3.1 Bile acid accumulation compromises anti-PD-1 efficacy in CRC

**Fig. 1:**
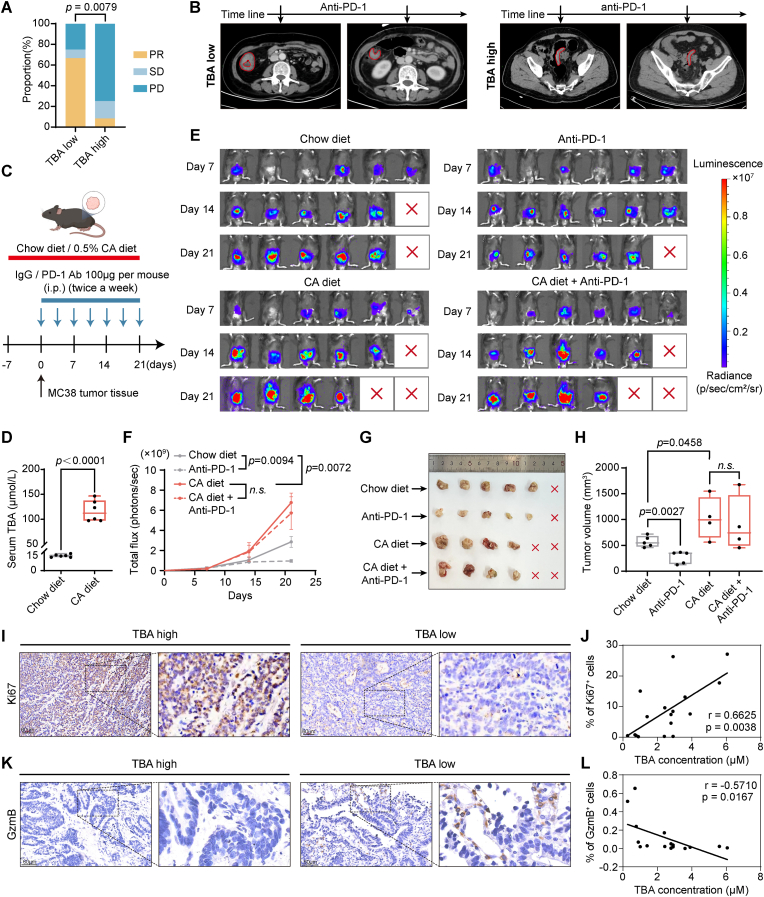
**Increased TBA concentration is significantly associated with low responsiveness to anti-PD-1 therapy. A,** The proportion of CRC patients with high and low TBA levels who exhibit different responsiveness to immunotherapy. PR: Partial response; SD: Stable disease; PD: Progressive disease. **B,** Representative CT scan images showing that CRC patients with low TBA levels responded sensitively to anti-PD-1 treatment, while patients with high TBA levels showed no response. Images of the primary tumor are delineated by red lines. **C,** Experimental scheme. C57BL/6 mice were fed with chow or 0.5% CA diet for 7 days, and MC38-luc tumor fragments were orthotopic implanted in the colon, while mice were administrated anti-PD-1 at the indicated time points. **D,** Concentration of total bile acids in serum of mice after chow or 0.5% CA diet feeding for 7 days (*n* = 6). **E** and **F,** Total flux (photons/sec) (**E**) and tumor bioluminescence (**F**) post 7, 14, 21 days of MC38-luc tumor fragments implantation. Images captured and total flux measured by IVIS following i. p. Injection of luciferin-D (*n* = 4-5). **G** and **H,** Representative images of tumor in colon (**G**) and tumor volume (**H**) after MC38 tumor fragments orthotopic implantation for 21 days (*n* = 4-5). **I** and **J,** Representative Ki67 IHC staining from CRC patients with high and low TBA levels and correlation analysis of the proportion of positive cells in Ki67 with TBA concentration (*n* = 17). **K** and **L,** Representative GzmB IHC staining from CRC patients with high and low TBA levels and correlation analysis of the proportion of positive cells in GzmB with TBA concentration (*n* = 17). *P*-value was assessed by Wilcoxon Rank-Sum Test (**A**). Data are presented as dot plots with fitted regression lines. The *r* and *p* represent the Pearson correlation coefficient and the two-tailed *p*-value, respectively (**J** and **L**). Data are presented as mean ± SEM, *p*-value was assessed by Student's *t*-test (**D**, **F**, and **H**).

Given the substantial heterogeneity in clinical responses to anti-PD-1 immunotherapy among CRC patients, we sought to evaluate whether serum bile acid levels are associated with therapeutic efficacy. To this end, we analyzed the correlation between serum total bile acid (TBA) concentrations and therapeutic responsiveness in a cohort of CRC patients receiving anti-PD-1 therapy. Patients were stratified into TBA^lo^ (bottom quartile, 0-25%) and TBA^hi^ (top quartile, 75–100%) cohorts based on their serum TBA levels. Wilcoxon rank-sum test analysis revealed that the objective response rate was significantly lower in the TBA^hi^ group than in the TBA^lo^ group (Fig. 1A). Computed tomography assessment further corroborated this disparity, while TBA^lo^ patients achieved sustained regression of primary tumors following treatment, TBA^hi^ patients retained a substantial burden of residual disease (Fig. 1B). These clinical data indicate that elevated systemic bile acid levels are strongly associated with low responsiveness to anti-PD-1 therapy in CRC.

To experimentally validate the impact of bile acid levels on the efficacy to anti-PD-1 therapy *in vivo*, we established an orthotopic CRC model in C57BL/6 mice and induced bile acid elevation using a cholic acid (CA)-supplemented diet (Fig. 1C). Following seven consecutive days of dietary intervention, serum TBA levels were significantly increased (Fig. 1D). Notably, while orthotopic tumors exhibited profound sensitivity to anti-PD-1 under chow diet conditions, the CA-induced high bile acid milieu rendered tumors refractory to treatment, as evidenced by sustained bioluminescence (IVIS) signals and the lack of a significant reduction in endpoint tumor volumes (Fig. 1E–H), demonstrating that an elevated bile acid environment significantly compromise the anti-tumor potency of anti-PD-1.

We further assessed whether elevated bile acid levels are linked to clinicopathological features of aggressive disease. Upon stratification of CRC patients based on serum TBA concentrations, immunohistochemical analysis revealed a significant positive correlation between TBA levels and the intratumoral expression of the proliferation marker Ki67. Conversely, expression of the cytotoxic effector molecule Granzyme B (GzmB) exhibited a significant inverse correlation with TBA levels (Fig. 1I–L).

Collectively, these data consistently demonstrate that an elevated bile acid milieu is significantly associated with refractoriness to anti-PD-1 therapy in CRC, supporting bile acid dysregulation as a clinically relevant metabolic feature linked to impaired immunotherapeutic efficacy.

### 3.2 CA diet-induced bile acid accumulation remodels the tumor immune microenvironment and impairs CD8^+^ T cell antitumor function

**Fig. 2:**
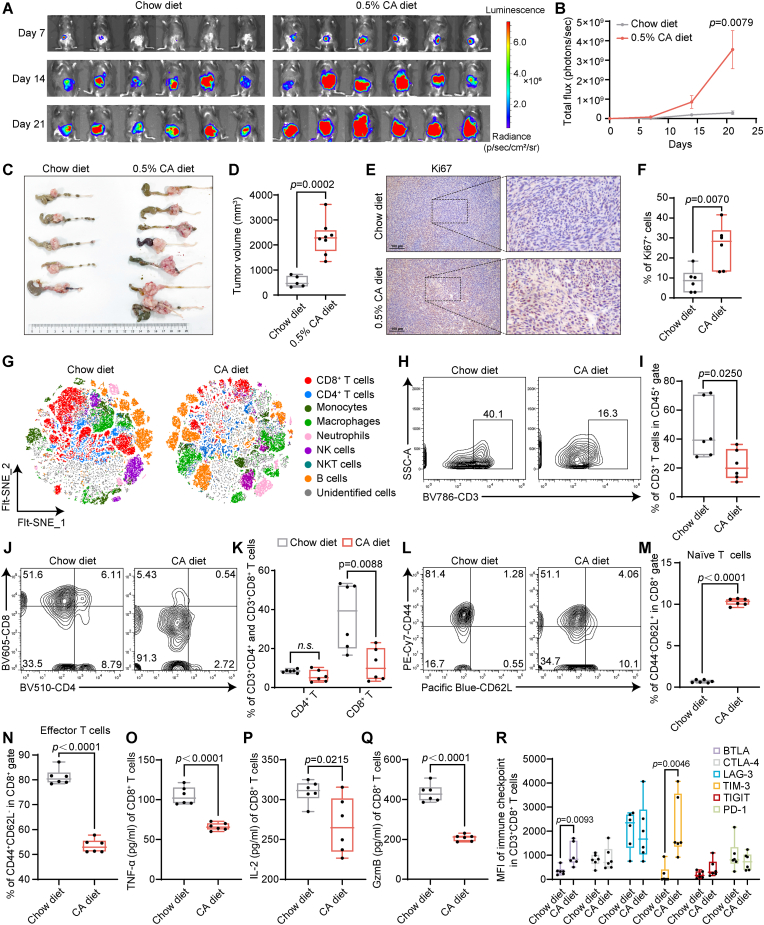
The CA diet reshapes the tumor immune microenvironment and impairs the numbers and functionality of intratumoral CD8^+^ T cells. **A** and **B,** Total flux (photons/sec) (**A**) and tumor bioluminescence (**B**) post 7, 14, 21 days of MC38-luc tumor fragments implantation. Images captured and total flux measured by IVIS following i. p. Injection of luciferin-D (*n* = 6). **C** and **D,** Representative images of tumor in colon (**C**) and tumor volume (**D**) in chow diet-fed (*n* = 5) and 0.5% CA diet-fed mice (*n* = 8) after MC38 tumor fragments orthotopic implantation for 21 days. **E** and **F,** Representative Ki67 IHC staining (**E**) and quantification (**F**) from mice tumor tissue on the 21st day after MC38 tumor fragments orthotopic implantation. **G,** Flt-SNE analysis of flow cytometry data from tumor-infiltrating CD45^+^ T cells from MC38 orthotopic tumor which fed with chow and 0.5% CA diet. Gating strategy is presented in Fig. S1 H and I, Representative flow cytometry (**H**) and quantitative data (**I**) of the percentage of CD3^+^ in CD45^+^ T cells isolated from MC38 orthotopic tumor tissue (*n* = 6). **J** and **K,** Representative flow cytometry (**J**) and quantitative data (**K**) of the percentage of CD3^+^CD4^+^ and CD3^+^CD8^+^ T cells isolated from MC38 orthotopic tumor (*n* = 6). **L**-**N,** Representative flow cytometry (**L**) and quantitative data (**M, N**) of the percentage of Naïve T cells (CD44^−^CD62l^+^) and effector T cells (CD44^+^CD62l^−^) in CD8^+^ T cells isolated from MC38 orthotopic tumor (*n* = 6). **O-Q,** Quantification of TNF-α (**O**), IL-2 (**P**) and GzmB (**Q**) secretion by CD8^+^ TILs after *ex vivo* αCD3/αCD28 stimulation (*n* = 6). **R,** Mean fluorescence intensity (MFI) of immune checkpoints (BTLA, CTLA-4, LAG-3, TIM-3, TIGIT, PD-1) expression on CD8^+^ TILs (*n* = 6). TIL, tumor-infiltrating lymphocyte. Data are presented as mean ± SEM, *p*-value was assessed by Student's *t*-test.

To delineate how dysregulated bile acid metabolism remodels CRC immune microenvironment, we performed a systematic immune profiling analysis in an orthotopic CRC model under CA dietary intervention. Consistent with the tumor-promoting effect observed above, CA diet significantly accelerated tumor growth, as reflected by enhanced bioluminescence IVIS signals (Fig. 2A and B), increased endpoint tumor volumes (Fig. 2C and D), and elevated Ki67 expression (Fig. 2E and F). We therefore next focused on whether this high bile acid state impairs antitumor immunity within the tumor microenvironment (TME). Using unbiased multidimensional flow cytometry combined with t-Distributed Stochastic Neighbor Embedding (t-SNE) analysis (Fig. 2G; Supplementary Fig. S1; Supplementary Fig. S2A). Compared to the chow diet, CA diet led to a marked suppression of total CD45^+^ leukocyte infiltration in the TME (Supplementary Fig. S2B and C). Specifically, among the immune subsets profiled, the T cell compartment exhibited the most profound decline, which was predominantly manifested as a significant decrease of CD8^+^ T cells, whereas the abundance of CD4^+^ T cells remained unchanged (Fig. 2H–K). Furthermore, decreases of varying magnitude in neutrophil and NKT cell frequencies were also observed (Supplementary Fig. S2D–N). These findings underscore that CA-induced bile acid accumulation preferentially restricts infiltration of CD8^+^ T cell, the dominant antitumor effector population in TME.

The transition of naïve T cells into an effector state upon antigen stimulation is a pivotal process for sustained antitumor immunity [20]. Further immunophenotyping analysis revealed that CA administration significantly altered the differentiation trajectory of tumor-infiltrating T cells, manifested by an accumulation of naïve T cells (CD44^−^CD62L^+^) and a concomitant reduction in effector T cells (CD44^+^CD62L^−^). This differentiation blockade was particularly pronounced in the CD8^+^ compartment, while milder alterations were observed in CD4^+^ T cells (Fig. 2L–N; Supplementary Fig. S2O–T). Consistent with this impeded differentiation, tumor-infiltrating CD8^+^ T cells from CA-fed mice displayed reduced production of TNF-α and IL-2, together with diminished GzmB expression (Fig. 2O–Q), indicating a marked loss of cytotoxic function.

Given the established relationship between immune checkpoint expression and efficacy of immunotherapy [21], we next characterized the checkpoint expression profile of tumor-infiltrating T cells. Notably, CA administration significantly upregulated the expression of TIM-3, a canonical marker of terminal exhaustion, specifically on CD8^+^ T cells, whereas checkpoint expression on CD4^+^ T cells remained largely unchanged (Fig. 2R; Supplementary Fig. S2U). Immunohistochemical analysis further corroborated these findings, revealing diminished CD8 expression alongside increased TIM-3 and BTLA expression in CA-treated tumors. To determine whether this checkpoint upregulation was driven by extrinsic signals, we assessed the expression levels of their respective ligands. However, the levels of Galectin-9 and TNFRSF14 were not significantly altered, suggesting that the bile acid induced immunosuppressive state is primarily mediated by intrinsic checkpoint alterations within CD8^+^ T cells rather than ligand upregulation in the TME (Supplementary Fig. S3).

Beyond the TME, we assessed whether CA diet-induced bile acid elevation impacts systemic immune homeostasis. Flow cytometric profiling of peripheral blood leukocytes in orthotopic CRC mice revealed that CA administration significantly reduced the frequency of circulating CD8^+^ T cells without affecting CD4^+^ T cells (Supplementary Fig. S4). Furthermore, distinct alterations were also noted in monocyte, neutrophil, and macrophage populations, reflecting a broader impact of elevated bile acids on systemic immune composition.

Collectively, these results show that CA-induced bile acid accumulation remodels the CRC immune microenvironment into an immunosuppressive state characterized by reduced CD8^+^ T cell infiltration, impaired effector differentiation, weakened cytotoxicity, and enhanced exhaustion, thereby providing an immune basis for the diminished efficacy of anti-PD-1 therapy.

### 3.3 Bile acid accumulation impairs anti-PD-1 efficacy through CD8^+^ T cell-dependent immune dysfunction and terminal exhaustion

**Fig. 3:**
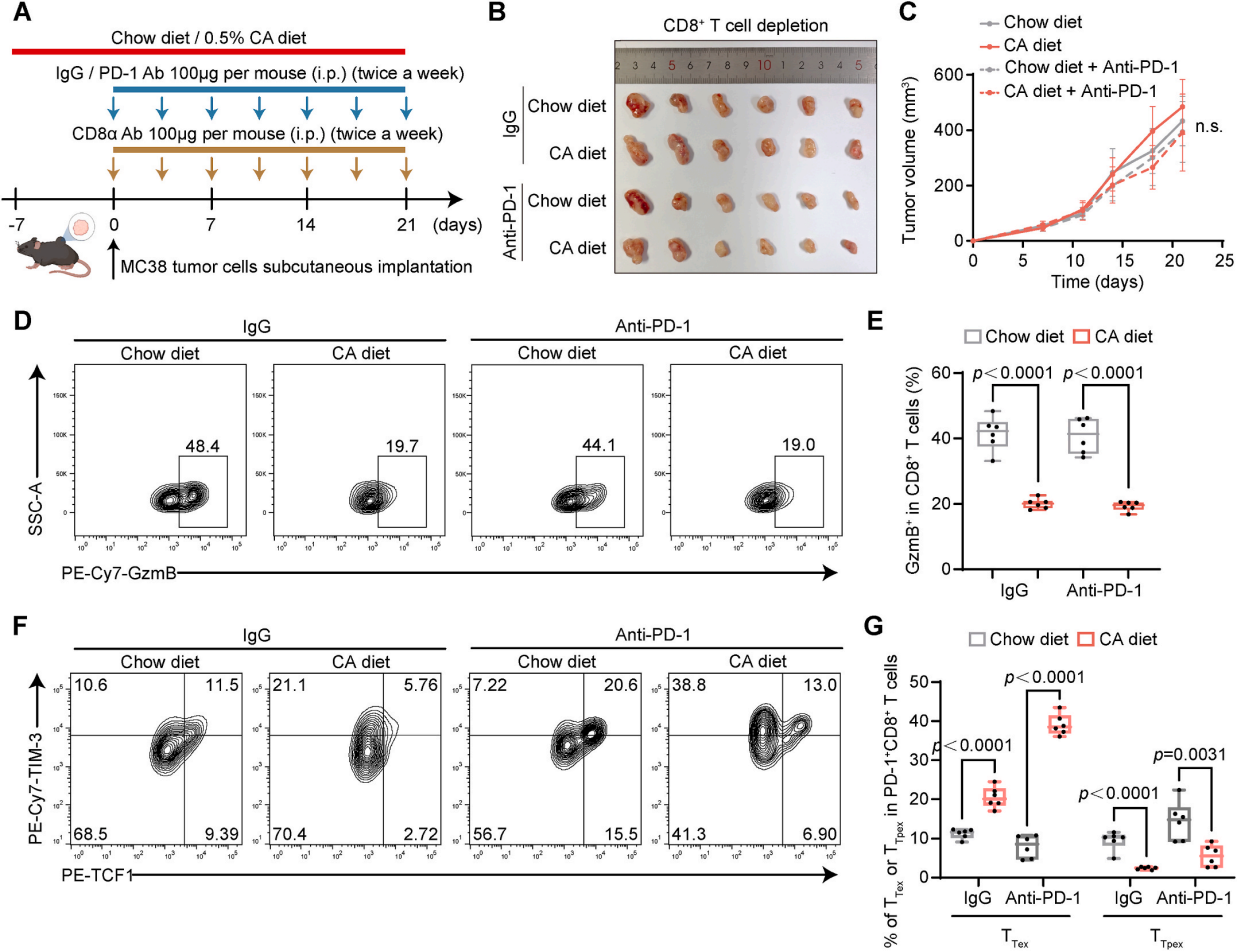
**Bile acid accumulation promotes CD8^+^ T cell exhaustion and impairs anti-PD-1 efficacy. A,** Experimental scheme. C57BL/6 mice were fed with chow or 0.5% CA diet for 7 days, subcutaneous implantation of MC38 tumor cells was performed, while MC38 tumor-bearing mice were administrated anti-CD8α and PD-1 blockade at the indicated time points. **B** and **C,** Representative images of subcutaneous tumor (**B**) and tumor volume (**C**) in MC38 tumor-bearing mice with CD8^+^ T cell depletion after chow or 0.5% CA diet feeding and under PD-1 blockade for 21 days (*n* = 7). **D** and **E,** Representative flow cytometry (**D**) and quantitative data (**E**) of the percentage of GzmB^+^ in CD8^+^ T cells isolated from MC38 orthotopic tumor model after chow or 0.5% CA diet feeding with or without anti-PD-1 therapy (*n* = 6). **F** and **G,** Representative flow cytometry (**F**) and quantitative data (**G**) of the percentage of T_Tex_ and T_Tpex_ in CD8^+^ T cells isolated from MC38 orthotopic tumor model after chow or 0.5% CA diet feeding with or without anti-PD-1 therapy (*n* = 6). Data are presented as mean ± SEM, *p*-value was assessed by Student's *t*-test.

Based on the observation that CA-diet compromises CD8^+^ T cell abundance and function within TME, we sought to determine whether impaired anti-PD-1 efficacy under high bile acid conditions depends specifically on CD8^+^ T cells. To address this, we administered CD8-neutralizing antibodies to deplete CD8^+^ T cells in MC38 tumor-bearing mice and compared chow diet and CA diet groups with or without PD-1 blockade (Fig. 3A). Notably, under CD8-depleted conditions, tumor growth was comparable between the chow diet and CA diet groups in the control IgG treatment. Similarly, during anti-PD-1 treatment, the difference in tumor growth between chow diet and CA diet groups was largely abolished after CD8^+^ T cell depletion (Fig. 3B and C). These results indicate that both the tumor-promoting effect of CA diet and the impaired response to anti-PD-1 therapy under high bile acid conditions are largely dependent on CD8^+^ T cell-mediated antitumor immunity.

Given that PD-1 blockade primarily rejuvenates progenitor exhausted T cells, we examined how high bile acid influence CD8^+^ T cell exhaustion status during anti-PD-1 therapy. In orthotopic CRC mice receiving anti-PD-1 therapy, CA diet significantly dampened GzmB expression in tumor-infiltrating CD8^+^ T cells (Fig. 3D and E). Importantly, immunophenotyping of the exhaustion hierarchy revealed that bile acid favored a transition toward a terminally exhausted phenotype (TIM-3^+^TCF1^−^PD-1^+^), accompanied by a corresponding reduction in the progenitor exhausted subset (TIM-3^−^TCF-1^+^PD-1^+^) [22,23] (Fig. 3F and G). These data suggest that a high bile acid state enforces a terminal differentiation program in CD8^+^ T cells, a detrimental effect that persists even during PD-1 blockade, thereby limiting its efficacy. Together, these findings identify CD8^+^ T cell dysfunction and terminal exhaustion as key immunological determinants of bile acid-induced resistance to anti-PD-1 therapy in CRC.

### 3.4 Deoxycholic acid is the key bile acid species driving CD8^+^ T cell terminal exhaustion in CRC

**Fig. 4:**
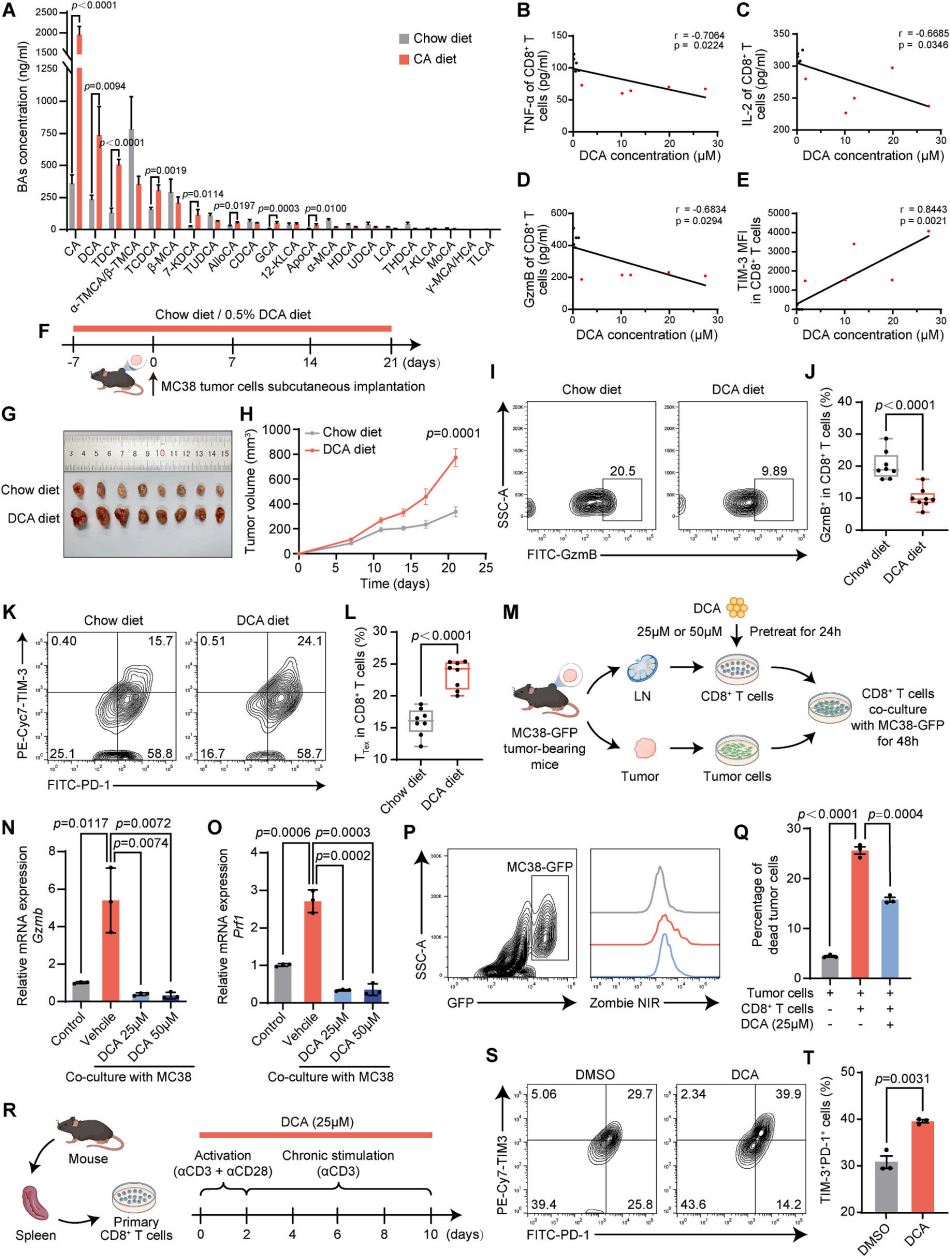
**Deoxycholic acid impairs CD8^+^ T cell function. A,** Bile acid profile analysis of different type bile acid concentration variations in the serum samples of chow diet-fed and 0.5% CA diet-fed MC38 tumor-bearing mice (*n* = 4-8). **B-D,** The correlation between DCA concentration and the supernatant TNF-α (**B**), IL-2 (**C**) and GzmB (**D**) concentrations in CD8^+^ TILs (*n* = 10). **E,** The correlation between DCA concentration and MFI of immune checkpoints (TIM-3) expression on CD8^+^ TILs (*n* = 10). **F,** Experimental scheme. C57BL/6 mice were fed with chow or 0.5% DCA diet for 7 days, subcutaneous implantation of MC38 tumor cells was performed. **G** and **H,** Representative images of subcutaneous tumor (**G**) and tumor volume (**H**) in MC38 tumor-bearing mice after chow or 0.5% DCA diet feeding for 21 days (*n* = 8). **I** and **J,** Representative flow cytometry (**I**) and quantitative data (**J**) of the percentage of GzmB^+^ in CD8^+^ T cells isolated from MC38 tumor tissue (*n* = 8). **K** and **L,** Representative flow cytometry (**K**) and quantitative data (**L**) of the percentage of T_Tex_ (TIM-3^+^PD-1^+^) in CD8^+^ T cells isolated from MC38 tumor tissue (*n* = 8). **M,** Schematic representation of CD8^+^ T cells (effector cells)-MC38-GFP (target cells) co-culture system. Axillary draining lymph nodes-derived CD8^+^ T cells pretreated with DCA obtained from MC38-GFP tumor-bearing mice were cultured with autologous MC38-GFP tumor cells at a ratio of 10:1 for 48h. **N** and **O,***Gzmb* (**N**) and *Prf1* (**O**) mRNA level detection by qRT-PCR in CD8^+^ T cells pretreated with 25 and 50 μM DCA which were collected from supernatant of co-culture system (*n* = 3). **P** and **Q,** Representative flow cytometry (**P**) and quantitative data (**Q**) of Zombie-NIR positive cells among MC38-GFP cells in co-culture system. GFP/Zombie-NIR double positive cells are considered as dead or dying target cells (*n* = 3). **R,** Schematic representation of the *ex vivo* chronic stimulation model. **S** and **T,** Representative flow cytometry (**S**) and quantitative data (**T**) of the percentage of T_Tex_ (TIM-3^+^TCF1^−^PD-1^+^) and T_Tpex_ (TCF1^+^TIM-3^−^PD-1^+^) in primary mouse CD8^+^ T cells pretreated with 25 μM DCA (*n* = 3). Data are presented as dot plots with fitted regression lines. The *r* and *p* represent the Pearson correlation coefficient and the two-tailed *p*-value, respectively (**B-E**). Data are presented as mean ± SEM, p-value was assessed by Student's *t*-test (**H**, **J**, **L**, **N**, **O**, **Q** and **T**).

The bile acid pool is highly complex metabolic landscape, comprising multiple bile acid species generated through gut microbial biotransformation [24,25]. However, which specific bile acid species within this elevated bile acid milieu that predominantly drives CD8^+^ T cell dysregulation remains elusive. To address this, we performed bile acid profiling on serum samples from orthotopic CRC mice subjected to CA feeding. We observed that, following exclusion of exogenous CA, DCA emerged as the most significantly upregulated endogenous bile acid species (Fig. 4A). Further correlative analysis revealed that serum DCA concentrations were significantly inversely correlated with the expression of effector molecules (TNF-α, IL-2, and GzmB) in tumor-infiltrating CD8^+^ T cells, while showing a robust positive correlation with the terminal exhaustion marker TIM-3 (Fig. 4B–E), identifying DCA as a candidate mediator of bile acid-associated CD8^+^ T cell dysfunction.

To directly substantiate the *in vivo* impact of DCA, we subjected MC38 tumor-bearing mice to a DCA-supplemented diet (Fig. 4F). Relative to the chow diet group, DCA administration significantly accelerated tumor growth (Fig. 4G and H) and was accompanied by impaired CD8^+^ T cell function, as evidenced by a reduced proportion of GzmB-expressing CD8^+^ T cells within TME (Fig. 4I and J). In parallel, DCA diet markedly increased the frequency of terminally exhausted CD8^+^ T cells (Tex: PD-1^+^TIM3^+^) (Fig. 4K and L). These findings indicate that DCA compromises the cytotoxic capacity of tumor-infiltrating CD8^+^ T cells and facilitates their transition into a state of terminal exhaustion.

We next asked whether DCA directly acts on CD8^+^ T cells. In primary murine CD8^+^ T cells, DCA treatment dose-dependent suppressed the expression of TNF-α, GzmB, and Perforin transcripts (Supplementary Fig. S5A–C). To better recapitulate the tumor-immune interplay characteristic of the TME, we established a co-culture system comprising CD8^+^ T cells isolated from the axillary lymph nodes of MC38-GFP tumor-bearing mice and autologous MC38-GFP tumor cells (Fig. 4M). Within this co-culture system, DCA treatment significantly inhibited the secretion of GzmB and Perforin by CD8^+^ T cells in a dose-dependent manner (Fig. 4N and O). Furthermore, assessment using a LIVE/DEAD viability assay demonstrated that DCA significantly impaired the cytolytic capacity of CD8^+^ T cells against autologous tumor targets (Fig. 4P and Q).

Importantly, utilizing an *in vitro* chronic stimulation model driven by continuous anti-CD3 antibody [26] (Fig. 4R), we observed that DCA treatment significantly facilitated the acquisition of a terminal exhaustion phenotype in CD8^+^ T cells, manifested by a marked elevation in the frequency of TIM-3^+^PD-1^+^ cells (Fig. 4S and T). Collectively, these findings identify DCA as a key bile acid species that directly impairs CD8^+^ T cell effector function and drives terminal exhaustion, thereby linking bile acid dysregulation to defective antitumor immunity in CRC.

### 3.5 DCA impairs mitochondrial oxidative metabolism and disrupts mitochondrial fitness in CD8^+^ T cells

**Fig. 5:**
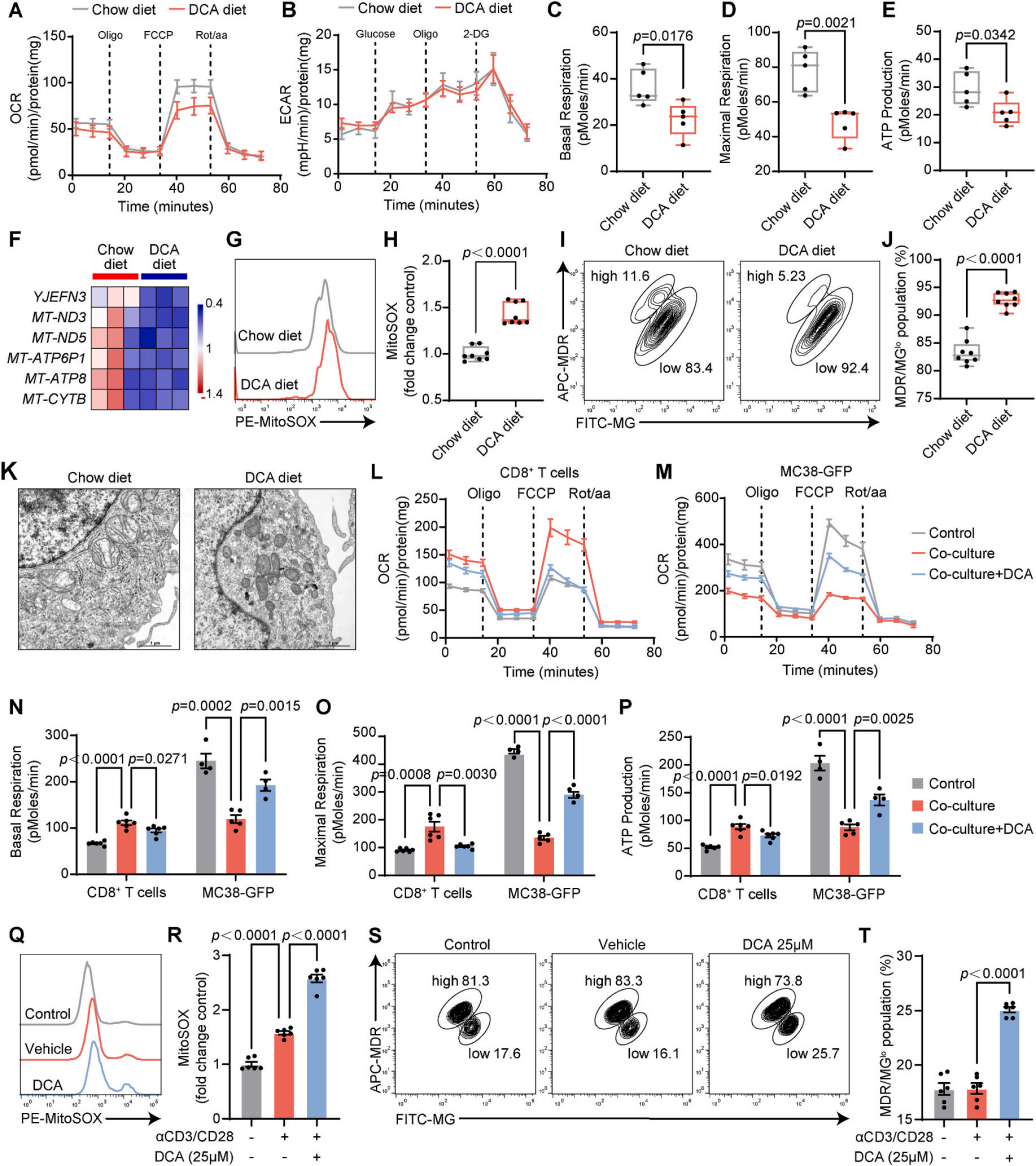
**DCA induces metabolic reprogramming and impairs mitochondrial function in CD8^+^ T cells. A** and **B,** Extracellular flux analysis of oxygen consumption rate (OCR) (**A**) and extracellular acidification rate (ECAR) (**B**) of tumor-infiltrating CD8^+^ T cells, which were isolated from chow or 0.5% DCA diet-fed MC38 tumor bearing mice (*n* = 5-6). **C-E,** Basal respiration (**C**), maximal respiration (**D**) and ATP production (**E**) of tumor-infiltrating CD8^+^ T cells (*n* = 5). **F,** Heatmap showing the normalized expression of genes for tumor-infiltrating CD8^+^ T cells, which were isolated from chow or 0.5% DCA diet-fed MC38 tumor bearing mice (*n* = 3). **G** and **H,** Representative flow cytometry (**G**) and quantitative data (**H**) of mitochondrial ROS accumulating levels measured by MitoSOX staining in tumor-infiltrating CD8^+^ T cells, which were isolated from chow or 0.5% DCA diet-fed MC38 tumor bearing mice (*n* = 8). **I** and **J,** On the basis of the ratio of MDR to MG, two distinct populations of CD8^+^ T cells were determined as MDR/MG^hi^ (upper left) and MDR/MG^lo^ (lower right) using flow cytometry (**I**), followed by the quantitative data (**J**) of the percentage of MDR/MG^lo^ populations in tumor-infiltrating CD8^+^ T cells, which were isolated from chow or 0.5% DCA diet-fed MC38 tumor bearing mice (*n* = 8). **K,** Representative electron microscope images for mitochondrion of tumor-infiltrating CD8^+^ T cells, which were isolated from chow or 0.5% DCA diet-fed MC38 tumor bearing mice. **L** and **M,** Extracellular flux analysis of oxygen consumption rate (OCR) of CD8^+^ T cells (**L**) (*n* = 7) and MC38-GFP tumor cells (**M**) (*n* = 4-5) in co-culture system (Fig. 4M). **N–P,** Basal respiration (**N**), maximal respiration (**O**) and ATP production (**P**) of CD8^+^ T cells (*n* = 6) and MC38-GFP tumor cells (*n* = 4-5) in co-culture system (Fig. 4M). **Q** and **R,** Representative flow cytometry (**Q**) and quantitative data (**R**) of mitochondrial ROS accumulating levels in primary mouse CD8^+^ T cells pretreated with 25 μM DCA for 24h and stimulated with αCD3/CD28 for 6h (*n* = 6). **S** and **T,** Representative flow cytometry (**S**) and quantitative data (**T**) of the percentage of MDR/MG^lo^ populations in primary mouse CD8^+^ T cells pretreated with 25 μM DCA for 24h and stimulated with αCD3/CD28 for 6h (*n* = 6). Data are presented as mean ± SEM, *p*-value was assessed by Student's *t*-test.

Building on our observation that DCA drives T cell terminal exhaustion, and given that metabolic reprogramming is a fundamental driver of T cell fate [27,28], we investigated whether DCA exerts its effect by reshaping the metabolic state of CD8^+^ T cells. For this purpose, we performed Seahorse extracellular flux analyses to measure oxygen consumption rate (OCR) and extracellular acidification rate (ECAR) as indicators of oxidative phosphorylation (OXPHOS) and aerobic glycolysis, respectively. DCA administration selectively suppressed OXPHOS in tumor-infiltrating CD8^+^ T cells, whereas aerobic glycolysis remained largely unaffected (Fig. 5A and B). Specifically, this metabolic defect was characterized by marked reductions in basal respiration, maximal respiration, and ATP production, while basal glycolysis and glycolytic capacity remained comparable to controls (Fig. 5C–E; Supplementary Fig. S5D and E). Consistent with these bioenergetic defects, bulk RNA-seq analysis revealed a significant downregulation of genes encoding core components of the mitochondrial respiratory chain in tumor-infiltrating CD8^+^ T cells from DCA-treated mice (Fig. 5F), which was further confirmed by qRT-PCR (Supplementary Fig. S5F–I). Together, these data indicate that DCA preferentially suppresses mitochondrial oxidative metabolism in CD8^+^ T cells.

Because impaired OXPHOS can promote electron leakage from the electron transport chain and consequent mitochondrial reactive oxygen species (mtROS) accumulation, a hallmark of mitochondrial dysfunction [29,30]. We next examined whether DCA induced mitochondria metabolic suppression was accompanied by mitochondrial damage. Indeed, tumor-infiltrating CD8^+^ T cells from DCA-fed mice exhibited significantly increased mitochondrial reactive oxygen species (mtROS) levels (Fig. 5G and H), consistent with impaired electron transport chain activity. We then assessed whether this metabolic defect was associated with loss of mitochondrial fitness and structural damage. To this end, we assessed mitochondrial membrane potential relative to mitochondrial mass by staining with MitoTracker Deep Red (MDR) and MitoTracker Green (MG). Notably, the ratio of MDR to MG, which is an indicator of mitochondrial fitness by reflecting mitochondrial activity per mitochondrial mass [16], was significantly reduced in DCA group, manifested by a higher frequency of CD8^+^ T cells with depolarized and dysfunctional mitochondrial phenotype (MDR/MG^lo^) (Fig. 5I and J). More importantly, transmission electron microscopy (TEM) provided ultrastructural confirmation of these defects. Specifically, mitochondria in DCA-treated CD8^+^ T cells exhibited damaged phenotypes, including disrupted cristae structures and declined cristae number and integrity (Fig. 5K). These findings indicate that DCA impairs mitochondrial fitness and induces structural mitochondrial damage in CD8^+^ T cells. The accumulation of depolarized mitochondria has been closely linked T cells to exhaustion [19,31], establishing a metabolic basis for DCA-associated programmed terminal exhaustion of tumor-infiltrating T cells.

To determine how DCA affects CD8^+^ T cell metabolic competence within a competitive tumor environment, we utilized an *in vitro* co-culture system. Co-culture with MC38 tumor cells significantly potentiated OXPHOS in CD8^+^ T cells, indicative of metabolic demands triggered by tumor antigenic stimulation. Strikingly, DCA treatment markedly inhibited this antigen-driven metabolic surge (Fig. 5L). Conversely, within the tumor cell compartment, co-culture with CD8^+^ T cells compromised tumor OXPHOS, whereas DCA treatment partially rescued the mitochondrial respiratory activity of tumor cells (Fig. 5M). Detailed analysis of metabolic parameters revealed that DCA significantly diminished basal respiration, maximal respiration, and ATP production in co-cultured CD8^+^ T cells, while exerting converse metabolic effects on tumor cells (Fig. 5N–P). These data suggest that DCA reshapes the balance of metabolic competition within the tumor-immune microenvironment, favoring tumor metabolic dominance at the expense of CD8^+^ T cell metabolic fitness. To clarify whether these mitochondrial perturbations were a direct consequence of DCA on CD8^+^ T cells rather than an indirect effect of the complex TME, we utilized an isolated murine primary CD8^+^ T cell system. DCA treatment recapitulated the mitochondrial functional impairments observed *in vivo*, leading to increased mtROS accumulation (Fig. 5Q and R) and a higher frequency of MDR/MG^lo^ population of CD8^+^ T cells (Fig. 5S and T). These data indicate that the mitochondrial defects induced by DCA in CD8^+^ T cells are largely cell-intrinsic rather than merely secondary to the tumor microenvironment.

### 3.6 DCA suppresses Parkin-dependent mitophagy to drive CD8^+^ T cell terminal exhaustion

**Fig. 6:**
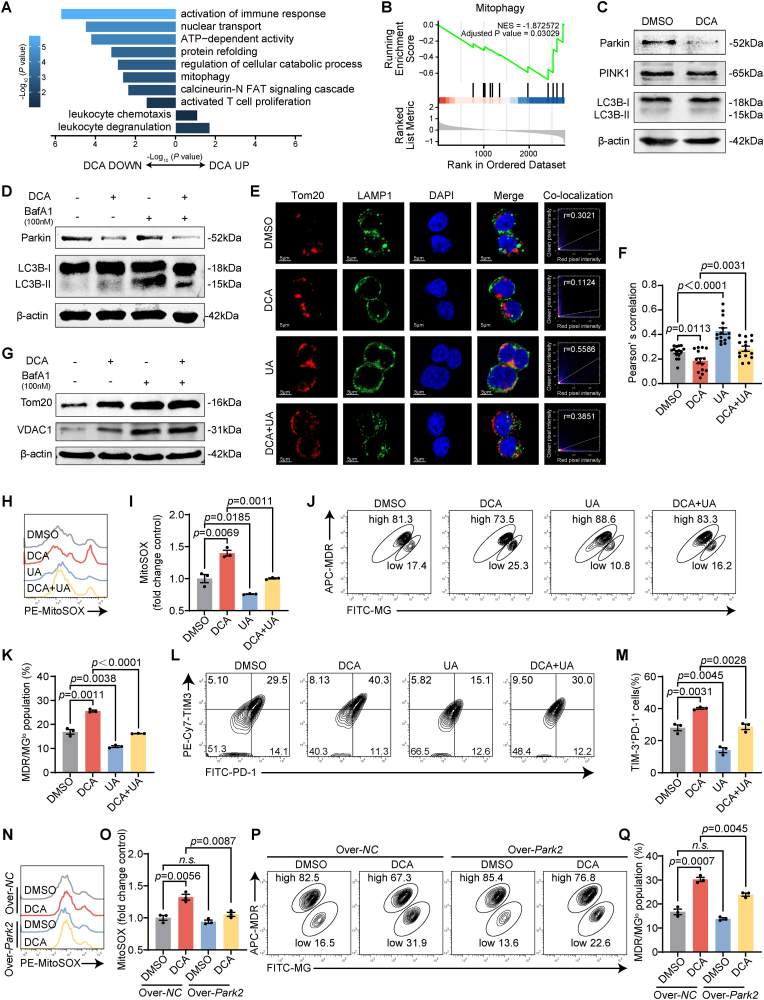
**DCA inhibits Parkin-dependent mitochondrial autophagy. A,** GO enrichment analysis of DEGs between tumor-infiltrating CD8^+^ T cells, which were isolated from chow or 0.5% CA diet-fed MC38 tumor bearing mice. **B,** GSEA of mitophagy signaling pathway in tumor-infiltrating CD8^+^ T cells, which were isolated from chow or 0.5% CA diet-fed MC38 tumor bearing mice. NES, normalized enrichment score. **C,** Representative images of Parkin, PINK, LC3B-Ⅰ, LC3B-Ⅱ and β-actin protein expression in whole-cell lysates extracted from primary mouse CD8^+^ T cells pretreated with DMSO or 25 μM DCA for 24h. **D,** Parkin, LC3B-Ⅰ, LC3B-Ⅱ and β-actin protein expression in CD8^+^ T cells pretreated with DMSO or 25 μM DCA for 24h with or without BafA1 (100 nM) for 4h. **E** and **F,** Representative immunofluorescence staining for mitophagy (**E**) and Pearson correlation analysis (**F**) in primary mouse CD8^+^ T cells pretreated with or without 25 μM DCA and 50 μM UA for 24h. Tom20 and LAMP1 were used to label mitochondria and lysosome, respectively. Staining for Tom20 (red), LAMP1 (green) and DAPI (blue). **G,** Tom20, VDAC1 and β-actin protein expression CD8^+^ T cells pretreated with DMSO or 25 μM DCA for 24h with or without BafA1 (100 nM) for 4h. **H** and **I,** Representative flow cytometry (**H**) and quantitative data (**I**) of mitochondrial ROS accumulating levels in primary mouse CD8^+^ T cells pretreated with or without 25 μM DCA and 50 μM UA for 24h (*n* = 3). **J** and **K,** Representative flow cytometry (**J**) and quantitative data (**K**) of the percentage of MDR/MG^lo^ populations in primary mouse CD8^+^ T cells pretreated with or without 25 μM DCA and 50 μM UA for 24h (*n* = 3). **L** and **M,** Representative flow cytometry (**L**) and quantitative data (**M**) of the percentage of T_Tex_ (TIM-3^+^PD-1^+^) in primary mouse CD8^+^ T cells pretreated with or without 25 μM DCA and 50 μM UA for 24h (*n* = 3). **N** and **O,** Representative flow cytometry (**N)** and quantitative data (**O**) of mitochondrial ROS accumulating levels in CD8^+^ T cells pretreated with or without 25 μM DCA for 24h after overexpressing *Park2* (*n* = 3). **P** and **Q,** Representative flow cytometry (**P**) and quantitative data (**Q**) of the percentage of MDR/MG^lo^ populations in CD8^+^ T cells pretreated with or without 25 μM DCA for 24h after overexpressing *Park2* (*n* = 3). Data are presented as mean ± SEM, *p*-value was assessed by Student's *t*-test.

For a more comprehensive understanding of the molecular mechanisms by which DCA compromises mitochondrial fitness in CD8^+^ T cells, we performed single-cell RNA sequencing (scRNA-seq) on tumor-infiltrating CD8^+^ T cells isolated from orthotopic CRC mice to profile the global transcriptomic alterations induced by DCA treatment. Gene Ontology (GO) enrichment analysis of differentially expressed genes (DEGs) revealed that ATP-dependent metabolic process and mitophagy pathway were significantly downregulated in the DCA group (Fig. 6A). Consistently, Gene Set Enrichment Analysis (GSEA) further substantiated the significant negative enrichment of the mitophagy gene set (Fig. 6B). These analyses identified suppressed mitophagy as a prominent transcriptional feature of DCA-exposed CD8^+^ T cells.

We next examined whether DCA directly perturbs mitophagy signaling in CD8^+^ T cells. Western blot analysis revealed that DCA treatment specifically downregulated the expression of Parkin, a pivotal regulator of ubiquitin-dependent mitophagy, whereas the levels of LC3B-Ⅰ and LC3B-Ⅱ remained largely unchanged (Fig. 6C), suggesting that DCA preferentially impairs the Parkin-dependent mitophagy axis. To further assess autophagic activity, CD8^+^ T cells were treated with Bafilomycin A1 (BafA1), which inhibits autophagosome-lysosome fusion and lysosomal acidification [32]. As expected, BafA1 treatment markedly increased LC3B-II accumulation, confirming effective inhibition of autophagic degradation. Notably, LC3B-II accumulation in response to BafA1 was significantly lower in DCA-treated cells, indicating that DCA suppresses autophagic flux (Fig. 6D). We then evaluated mitophagy by assessing mitochondrial-lysosomal interaction using immunofluorescence analysis of Tom20 and LAMP1. DCA exposure markedly reduced in Tom20-LAMP1 co-localization (Fig. 6E and F). Consistently, DCA increased the accumulation of the mitochondrial proteins Tom20 and VDAC1, and BafA1 further enhanced their accumulation (Fig. 6G), indicating impeded mitochondrial delivery to lysosomes and impaired mitophagy flux.

To further clarify upstream mechanisms, we performed a systematic mechanistic exclusion analysis of potential uptake and signaling pathways, including endocytosis, bile acid transporters, surface receptor involvement, extracellular pH, and structural analog effects. We found that none of these perturbations significantly affected intracellular DCA accumulation or reversed DCA-induced downregulation of Parkin, indicating that classical uptake and receptor-mediated mechanisms are not the primary drivers of DCA action in CD8^+^ T cells (Supplementary Fig. S6A–J). Moreover, intracellular DCA accumulation increased in an approximately linear manner with increasing extracellular concentrations without evidence of saturation within the tested range (Supplementary Fig. S6K), supporting a non-saturable, non-carrier-mediated uptake behavior.

To test whether restoring mitophagy could counteract DCA-induced mitochondrial defects, we performed rescue experiments using Urolithin A (UA), a well-characterized activator of Parkin-dependent mitophagy [33]. UA treatment robustly potentiated the co-localization of Tom20 and LAMP1, effectively counteracting the mitophagy suppression induced by DCA (Fig. 6E and F). In parallel, UA attenuated mtROS accumulation and restored mitochondrial fitness in DCA-exposed CD8^+^ T cells (Fig. 6H–K). Importantly, UA also substantially reduced the acquisition of the terminal exhaustion phenotype in DCA treatment (Fig. 6L and M).

While UA-mediated mitophagy activation successfully bypassed the DCA-induced metabolic block, we sought to confirm whether Parkin is the central molecular requirement for this effect. To this end, we overexpressed Parkin in CD8^+^ T cells. Strikingly, Parkin overexpression profoundly ameliorated DCA-induced mitochondrial dysfunction, characterized by the reduced mtROS accumulation and restoration of mitochondrial activity (Fig. 6N–Q). These data substantiate Parkin as a critical target through which DCA suppresses mitophagy and disrupts mitochondrial homeostasis in CD8^+^ T cells.

Collectively, these findings show that DCA suppresses Parkin-dependent mitophagy in CD8^+^ T cells, leading to mitochondrial dysfunction and promoting terminal exhaustion. Restoration of mitophagy, either pharmacologically or through Parkin overexpression, is sufficient to rescue mitochondrial homeostasis and alleviate DCA-induced CD8^+^ T cell dysfunction.

### 3.7 DCA promotes Parkin ubiquitination and proteasomal degradation

**Fig. 7:**
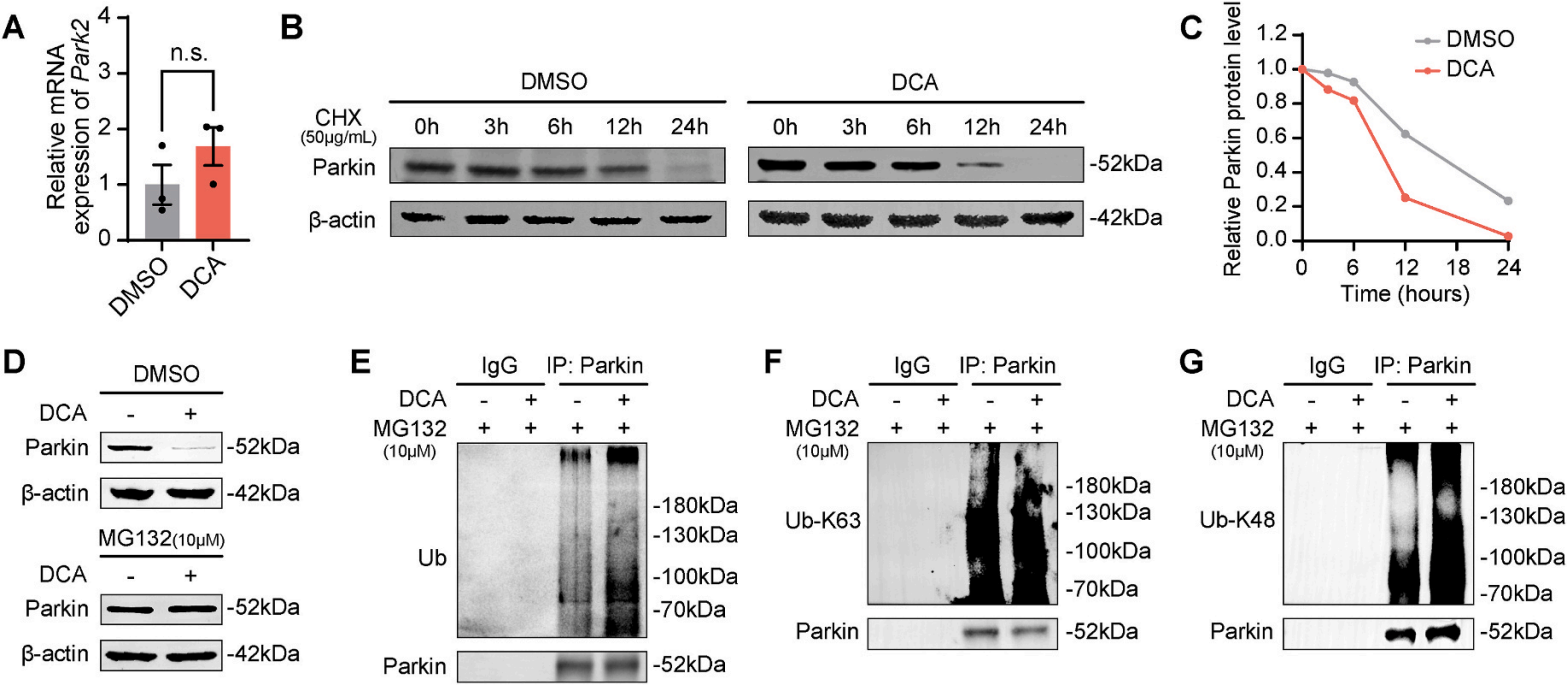
**Deoxycholic acid promotes Parkin ubiquitination and proteasomal degradation. A,***Park2* mRNA level detection by qRT-PCR in CD8^+^ T cells pretreated with 25 μM DCA for 24h (*n* = 3). **B** and **C,** DCA modulates the half-life of Parkin protein in CD8^+^ T cells (**B**) and quantitative analysis (**C**) of parkin protein in DMSO and DCA treated group by Western blot following treatment with 50 μg/mL CHX at various time points (0, 3, 6, 12 and 24h). **D,** Parkin expression in CD8^+^ T cells pretreated with DMSO or 25 μM DCA for 24h with or without 26S proteasome inhibitor MG132 (10 μM) for 6h. **E-G,** Total ubiquitinated (**E**), K63-linked ubiquitinated (**F**) and K48-linked ubiquitinated (**G**) parkin was immunoprecipitated in CD8^+^ T cells pretreated with or without 25 μM DCA for 24h using anti-Parkin antibody. Ub, ubiquitin. Data are presented as mean ± SEM, *p*-value was assessed by Student's *t*-test.

We further sought to elucidate the molecular mechanism underlying DCA-mediated downregulation of Parkin. While DCA significantly reduced Parkin protein levels (Fig. 6C), qRT-PCR analysis revealed no significant alterations in Parkin mRNA expression (Fig. 7A), indicating that DCA regulates Parkin primarily at the post-translational level. Given that Parkin stability is predominantly regulated by ubiquitin-proteasome system [34,35], we speculated that DCA may accelerate Parkin degradation by promoting its ubiquitination. To test this, we first investigated the effect of protein synthesis inhibitor cycloheximide (CHX) on the stability of Parkin protein under DCA treatment. While CHX treatment alone led to a gradual decrease in Parkin protein levels, the addition of DCA significantly accelerated its degradation rate, markedly shortening the half-life of the Parkin protein (Fig. 7B and C), indicating that DCA caused a decrease in Parkin protein stability. Then, to determine whether DCA-mediated Parkin downregulation occurs through a post-translational mechanism, CD8^+^ T cells were exposed to DCA in the presence or absence of the proteasome inhibitor MG132 (10 μM). Strikingly, MG132 treatment effectively blocked DCA-induced Parkin degradation, restoring its protein levels to near-basal conditions (Fig. 7D). Finally, we performed immunoprecipitation (IP) assays to evaluate Parkin ubiquitination following DCA treatment. Consistent with our hypothesis, DCA treatment significantly potentiated the ubiquitination of Parkin (Fig. 7E). We next examined the ubiquitin linkage pattern of Parkin. DCA did not affect K63-linked ubiquitination (Fig. 7F), but markedly increased K48-linked ubiquitination of Parkin (Fig. 7G), further supporting proteasome-dependent degradation of Parkin. Taken together, these findings demonstrate that DCA reduces Parkin protein abundance by promoting its ubiquitin-proteasome-dependent degradation, thereby impairing Parkin-dependent mitophagy and mitochondrial clearance in CD8^+^ T cells.

### 3.8 Pharmacological restoration of mitophagy restores anti-PD-1 efficacy under DCA exposure

**Fig. 8:**
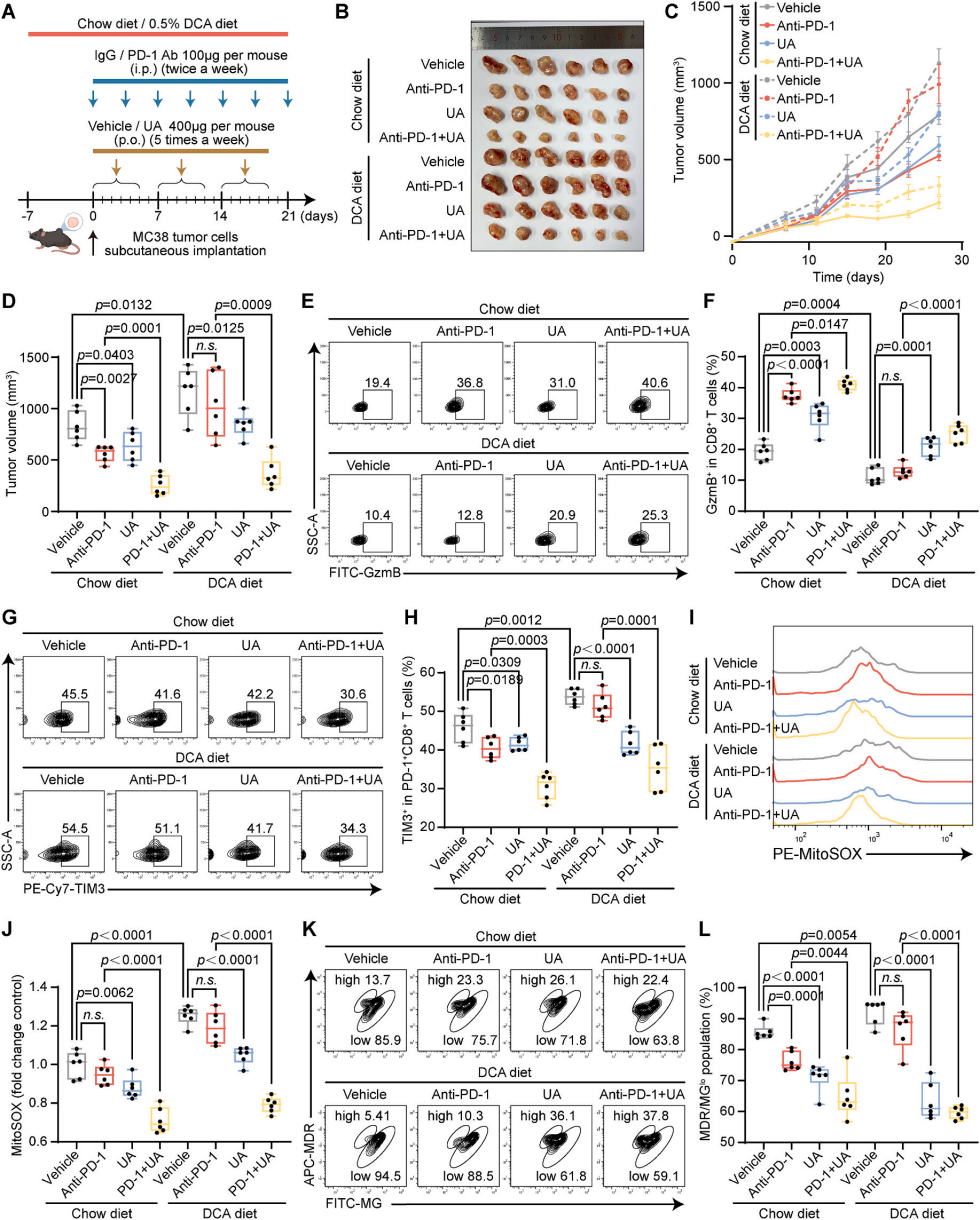
**Activation of mitophagy restores CD8^+^ T cell function and anti-PD-1 responsiveness under DCA exposure. A,** Experimental scheme. C57BL/6 mice were fed with chow or 0.5% DCA diet for 7 days, subcutaneous implantation of MC38 tumor cells was performed, while mice were administrated anti-PD-1 and UA at the indicated time points. **B-D,** Representative images of tumor in colon (**B**) and tumor volume (**C, D**) in MC38 tumor-bearing mice (*n* = 6). **E** and **F,** Representative flow cytometry (**E**) and quantitative data (**F**) of the percentage of GzmB^+^ in CD8^+^ T cells isolated from MC38 tumor tissue (*n* = 6). **G** and **H,** Representative flow cytometry (**G**) and quantitative data (**H**) of the percentage of TIM-3^+^PD-1^+^ in CD8^+^ T cells isolated from MC38 tumor tissue (*n* = 6). **I** and **J,** Representative flow cytometry (**I**) and quantitative data (**J**) of mitochondrial ROS accumulating levels in CD8^+^ T cells isolated from MC38 tumor tissue (*n* = 6). **K** and **L,** Representative flow cytometry (**K**) and quantitative data (**L**) of the percentage of MDR/MG^lo^ populations in CD8^+^ T cells isolated from MC38 tumor tissue (*n* = 6). Data are presented as mean ± SEM, *p*-value was assessed by Student's *t*-test.

Finally, to determine whether restoring mitophagy could reverse DCA-induced CD8^+^ T cell dysfunction and reinstate the efficacy of anti-PD-1 *in vivo*, we treated MC38 tumor-bearing mice with DCA in combination with the mitophagy activator UA and anti-PD-1 (Fig. 8A). While anti-PD-1 therapy significantly restricted tumor growth in mice fed with chow diet, this therapeutic benefit was largely abolished by DCA administration, confirming that DCA markedly impairs the antitumor efficacy of anti-PD-1. Notably, UA administration alone exhibited marginal anti-tumor activity; however, its combination with anti-PD-1 synergistically restored tumor control in DCA-exposed mice, yielding tumor volumes significantly lower than those in DCA plus anti-PD-1 group (Fig. 8B–D). These findings demonstrate that pharmacological activation of mitophagy is sufficient to recover anti-PD-1 efficacy under DCA exposure.

We next investigate whether this restoration of anti-PD-1 therapy sensitivity was attributable to functional recovery of tumor-infiltrating CD8^+^ T cells. In the context of DCA exposure, UA treatment significantly upregulated GzmB expression while diminishing the frequency of terminally exhausted CD8^+^ T cells. Importantly, the combination of UA and anti-PD-1 further potentiated effector function and significantly downregulated terminal exhaustion-associated markers (Fig. 8E–H). These findings indicate that UA-mediated restoration of mitophagy rescues DCA-induced terminal exhaustion of CD8^+^ T cells, thereby re-sensitizes them to anti-PD-1.

Consistent with these immunological improvements, UA treatment significantly alleviated DCA-induced mitochondrial defects in CD8^+^ T cells. Specifically, UA effectively abrogated the aberrant accumulation of mtROS and restored mitochondrial fitness, as evidenced by a decreased MDR/MG^lo^ ratio. These improvements in mitochondrial phenotypes were similarly observed following combinatorial treatment with UA and anti-PD-1 in DCA-exposed mice (Fig. 8I–L). Collectively, these results demonstrate that pharmacological activation of mitophagy reverses DCA-induced mitochondrial dysfunction, thereby recovering CD8^+^ T cell effector capacity and re-establishing responsiveness to anti-PD-1 therapy.

## 4 Discussion

Although anti-PD-1 therapy confers clinical benefits in a subset of patients with CRC, therapeutic responses remain highly heterogeneous. Even within MSI-H tumors, durable clinical benefit is not universal [36,37]. The determinants of this heterogeneity are complex, involving underappreciated non-genetic regulatory mechanisms. Our study identifies that the secondary bile acid DCA as a previously unrecognized metabolite determinant associated with low responsiveness to anti-PD-1 therapy in CRC. Mechanistically, DCA inhibits mitophagy in CD8^+^ T cells by promoting the ubiquitin-dependent degradation of Parkin, leading to the accumulation of dysfunctional mitochondria and drives CD8^+^ T cells toward terminal exhaustion, ultimately rendering PD-1 blockade ineffective. Collectively, these highlight DCA-driven disruption of mitochondrial homeostasis as a pivotal metabolic mechanism linking the tumor metabolic milieu to CD8^+^ T cell dysfunction and limited responsiveness to PD-1 blockade in CRC.

Previous studies have shown that secondary bile acids, particularly DCA, are significantly elevated in the feces of CRC patients and in high-risk populations [[38], [39], [40]]. In Apc^min/+^ mice, dietary supplementation with the CA (as a precursor of DCA) markedly promotes intestinal tumorigenesis, as corroborated by our orthotopic CRC model in which a CA-enriched diet significantly accelerated tumor progression [41]. Notably, bile acids do not function as isolated metabolites but instead comprise a highly complex and dynamic metabolic pool [25]. Distinct bile acid species exert pleiotropic effects on immune cells through receptor-dependent pathways, including FXR and TGR5 signaling, as well as receptor-independent mechanisms, collectively shaping tumor-immune interactions [15,[42], [43], [44], [45]]. However, despite accumulating evidence implicating bile acids are involved in CRC progression, whether and how bile acid dysregulation influences the efficacy of ICB by remodeling the immune microenvironment remains poorly understood. Integrating clinical analyses, we found that CRC patients with elevated total bile acid levels not only exhibit accelerated tumor progression but also significantly blunted responses to anti-PD-1 therapy.

Although previous work suggests that DCA inhibits CD8^+^ T cell activity by perturbing calcium signaling [15], this research does not account for the correlation between the efficacy of anti-PD-1 therapy and T cell immunity in a high-bile acid context. We showed that DCA exposure does not merely induce transient functional suppression but rather drives CD8^+^ T cells toward a state of terminal exhaustion. It is worth noting that this state of exhaustion persists despite anti-PD-1 intervention, implying that DCA-induced dysfunction is not solely dependent on the PD-1 pathway. This distinction is critical for understanding therapeutic failure, since although anti-PD-1 therapy exerts its antitumor effect primarily by reinvigorating progenitor exhausted T cells [46,47], DCA-induced drive toward terminal differentiation deplete this response pool.

Metabolic reprogramming dictates T cell fate and functional states, integrating bioenergetic and biosynthetic cues to regulate activation, effector capacity, and exhaustion [27,28,48]. Building on our previous observation that DCA interferes with CD4^+^ T cell metabolism [14], we here identified mitochondrial metabolism as the principal metabolic node through which DCA constrains CD8^+^ T cell function. We show that DCA profoundly suppresses mitochondrial respiration and induces structural and functional mitochondrial damage in CD8^+^ T cells, establishing a metabolic state incompatible with sustained effector activity. Notably, in addition to its direct metabolic inhibition on CD8^+^ T cells, DCA can also indirectly amplify its immunosuppressive effects by reshaping the metabolic competition landscape within the TME. Our co-culture analyses reveal that DCA restricts the mitochondrial metabolic capacity of CD8^+^ T cells while promoting the mitochondrial metabolic adaptability of tumor cells, suggesting that tumor cells may gain a relative metabolic advantage in high bile acid environments. Under such circumstances, the TME forms a competitive metabolic environment unfavorable for CD8^+^ T cell function, further exacerbating the metabolic constraints and exhaustion of T cells [49,50]. This metabolic "see-saw effect" may explain the heterogeneity of ICB efficacy in patients with different bile acid levels.

Mitochondrial homeostasis relies on a series of finely regulated quality control mechanisms [51,52], in which mitophagy plays a central role in maintaining the metabolic adaptability and functional integrity of immune cell mitochondria [16,[53], [54], [55]]. Our study demonstrates that DCA specifically disrupts the Parkin-dependent mitophagy pathway, leading to abnormal accumulation of damaged mitochondria. This not only triggers metabolic disorders and oxidative stress, but also impairs the metabolic plasticity required for T cells to maintain their effector state, thereby creating favorable conditions for their transition to a terminally exhausted phenotype [19,56,57].

At the molecular level, the protein stability of Parkin is precisely regulated by the ubiquitin-proteasome system. Studies have demonstrated that Parkin can be ubiquitinated by upstream E3 ligases and degraded via the proteasomal pathway [34,58]. Our research further reveals that DCA modulates Parkin levels primarily at the post-translational modification level by enhancing its K48-linked polyubiquitination and accelerating proteasome-dependent degradation, thereby limiting the functional availability of Parkin. This discovery further refines the link between bile acid metabolic signaling and mitochondrial quality control to the post-translational regulatory level. It also suggests that Parkin homeostasis may constitute one of the critical nodes where immunometabolism becomes susceptible to disruption under high bile acid conditions. Notably, although our ubiquitin proteomics analysis suggests that bile acid exposure also affect the ubiquitination of multiple protein substrates (data not shown), future studies are required to define these broader ubiquitination events and to investigate the upstream regulatory mechanisms responsible for DCA-induced Parkin ubiquitination.

In summary, our study identifies DCA as a key metabolic mediator of bile acid-associated impairment of anti-PD-1 efficacy in CRC. DCA disrupts CD8^+^ T cell mitochondrial homeostasis by impairing mitochondrial fitness and suppressing Parkin-dependent mitophagy, thereby promoting terminal exhaustion Pharmacological activation of mitophagy effectively rescues these metabolic defects and reinstates the efficacy of PD-1 blockade. These findings raise the possibility that elevated bile acid burden, particularly a DCA-enriched state, may help stratify CRC patients with poor immunotherapy responsiveness and support metabolic intervention as a rational strategy to enhance checkpoint blockade.

However, this study also has some limitations that merit additional exploration. First, while established DCA as a causal driver of T cell terminal exhaustion, the upstream microbial determinants responsible for DCA accumulation within the TME remain to be fully defined. Future interventions targeting bile acid-modifying bacterial populations to reshape the bile acid profile in TME at its source may emerge as a novel adjuvant strategy to enhance ICB efficacy. Secondly, elucidating the specific transmembrane transport mechanism of DCA into CD8^+^ T cells and identifying its key intracellular receptors will provide theoretical support for developing highly selective targeted inhibitors. This approach enables precise restoration of T cell mitochondrial fitness while minimizing potential interference with systemic metabolic homeostasis.

## Data availability

All other raw data generated in this study are available upon request from the corresponding author.

## Funding

This work was supported by the 10.13039/501100001809National Natural Science Foundation of China (82403258); the Haiyan Foundation of 10.13039/100014418Harbin Medical University Cancer Hospital (JJZD2024-03); the 10.13039/501100005046Natural Science Foundation of Heilongjiang Province (PL2024H162); the New Era Longjiang Excellent Master's and Doctoral Dissertations (LJYXL2023-078).

## CRediT authorship contribution statement

**Yang Gao:** Data curation, Formal analysis, Validation, Visualization, Writing – original draft. **Jiaxin Wang:** Data curation, Methodology, Software, Validation, Writing – original draft. **Shihui Lai:** Data curation, Formal analysis, Validation, Writing – review & editing. **Mingwei Li:** Validation. **Jiaqi Shi:** Writing – review & editing. **Xianjun Li:** Writing – review & editing. **Ying Guo:** Writing – review & editing. **Sainan Ma:** Investigation. **Xinxin Wang:** Investigation. **Shule Zhang:** Data curation, Validation. **Tianyu He:** Data curation, Validation. **Yu Hong:** Data curation, Validation. **Shuang Li:** Project administration, Supervision. **Caiqi Liu:** Project administration, Supervision. **Lijuan Cao:** Conceptualization, Supervision. **Haiping Hao:** Conceptualization, Supervision. **Chujie Ding:** Conceptualization, Funding acquisition, Project administration, Resources, Supervision, Validation, Writing – review & editing.

## Declaration of competing interest

The authors declare that they have no known competing financial interests or personal relationships that could have appeared to influence the work reported in this paper.

## Appendix A Supplementary data

Figure S1 Illustrative gating strategy for immune cells in TME.

Figure S2 **Related to**Fig. 2 A**,** Quantification of immune-cell composition based on the t-SNE-defined immune-cell populations. **B** and **C,** Representative flow cytometry (**B**) and quantitative data (**C**) of the percentage of CD45^+^ T cells isolated from MC38 orthotopic tumor tissue (*n* = 6). **D-I,** Representative flow cytometry (**D, F, H**) and quantitative data (**E, G, I**) of the percentage of monocytes (**E**, CD11b^+^), macrophages (**G**, CD11b^+^F4/80^+^) and neutrophils (**I**, CD11b^+^Ly6g^+^) among intratumoral CD45^+^ T cells (*n* = 6). **J-N,** Representative flow cytometry (**J, L**) and quantitative data (**K, M, N**) of the percentage of B cells (**K**, CD19^+^), NK cells (**M**, NK1.1^+^CD3^−^) and NKT (**N**, NK1.1^+^CD3^+^) among intratumoral CD45^+^ T cells (*n* = 6). **O-Q,** Representative flow cytometry (**O**) and quantitative data (**P, Q**) of the percentage of Naïve T cells (CD44^−^CD62l^+^) and effector T cells (CD44^+^CD62l^−^) in CD3^+^ T cells isolated from MC38 orthotopic tumor (*n* = 6). **R-T,** Representative flow cytometry (**R**) and quantitative data (**S, T**) of the percentage of Naïve T cells (CD44^−^CD62l^+^) and effector T cells (CD44^+^CD62l^−^) in CD4^+^ T cells isolated from MC38 orthotopic tumor (*n* = 6). **U,** Mean fluorescence intensity (MFI) of immune checkpoints (BTLA, CTLA-4, LAG-3, TIM-3, TIGIT, PD-1) expression on CD4^+^ TILs (*n* = 6). Data are presented as mean ± SEM, *p*-value was assessed by Student's *t*-test.

Figure S3 **Related to**Fig. 2 A-H**,** Representative IHC staining (**A**, **C**, **E**, **G**) and quantification (**B**, **D**, **F**, **H**) of CD8 and immune checkpoints (TIM3, PD-1, BTLA) and inhibitory receptors (Galectin-9, PD-L1, TNFRSF14) from mice tumor tissue on the 21st day after MC38 tumor fragments orthotopic implantation. Data are presented as mean ± SEM, *p*-value was assessed by Student's *t*-test.

Figure S4 **0.5% CA diet induces immune cells subsets change in PBMC of MC38 tumor-bearing mice. A-C,** Representative flow cytometry (**A**) and quantitative data (**B, C**) of CD45^+^ and CD3^+^ T cells in PBMC of MC38 tumor-bearing mice (*n* = 5). **D-F,** Representative flow cytometry (**D**) and quantitative data (**E, F**) of CD3^+^CD4^+^ and CD3^+^CD8^+^ T cells among CD45^+^ cells in PBMC of MC38 tumor-bearing mice (*n* = 5). **G-K,** Representative flow cytometry (**G, I**) and quantitative data (**H, J, K**) of B cells (**H**, CD19^+^), NK cells (**J**, NK1.1^+^CD3^−^) and NKT (**K**, NK1.1^+^CD3^+^) among CD45^+^ cells in PBMC of MC38 tumor-bearing mice (*n* = 5). **L-O,** Representative flow cytometry (**L**) and quantitative data (**M, N, O**) of the percentage of monocytes (**M**, CD11b^+^), macrophages (**N**, CD11b^+^F4/80^+^) and neutrophils (**O**, CD11b^+^Ly6g^+^) among CD45^+^ cells in PBMC of MC38 tumor-bearing mice (*n* = 5). Data are presented as mean ± SEM, *p*-value was assessed by Student's *t*-test.

Figure S5 **A-C,***Tnfα* (**A**), *Gzmb* (**B**) and *Prf1* (**C**) mRNA level detection by qRT-PCR in CD8^+^ T cells pretreated with 25 and 50 μM DCA for 24h and stimulated with αCD3/CD28 for 6h (*n* = 3). **D** and **E,** Glycolysis (**D**) and glycolytic capacity (**E**) of tumor-infiltrating CD8^+^ T cells (*n* = 5-6). **F–I,***Mt-nd3* (**F**), *Mt-nd5* (**G**), *Mt-atp8* (**H**) and *Mt-cytb* (**I**) mRNA level detection by qRT-PCR in CD8^+^ T cells, which were isolated from chow or 0.5% DCA diet-fed MC38 tumor bearing mice (*n* = 3). Data are presented as mean ± SEM, *p*-value was assessed by Student's *t*-test.

Figure S6 **DCA uptake mechanism in CD8^+^ T cells. A,** Quantification of intracellular DCA levels in CD8^+^ T cells treated with DMSO, DCA, or DCA plus Dynasore (80 μM) for 24h. **B–F,** Relative mRNA expression of bile acid transporters in hepatocytes, enterocytes, and CD8^+^ T cells. **G,** Western blot analysis confirming efficient knockdown of TGR5 and S1PR2. **H,** Western blot analysis of Parkin expression in CD8^+^ T cells treated with DMSO or DCA after transfection with si-*NC*, si-*Gpbar1* or *si-S1*pr*2*. **I,** Measurement of culture medium pH after DMSO or DCA treatment. **J,** Western blot analysis of Parkin expression after treatment with DMSO, DCA, CDCA, or UDCA. **K,** Quantification of intracellular DCA levels in CD8^+^ T cells after treatment with increasing concentrations of DCA. The R^2^ and *p* values were obtained by ordinary least-squares linear regression analysis (**K**). Data are presented as mean ± SEM, *p*-value was assessed by Student's *t*-test (**A-F,** and **I**).
