# Supplementary material for: Deoxycholic acid impairs mitochondrial fitness and suppresses mitophagy through Parkin ubiquitination driving CD8+ T cell Exhaustion in Colorectal Cancer
Source: Redox Biol. 2026 Jul 25;96:104322. doi: 10.1016/j.redox.2026.104322 (PMC13450440; doi:10.1016/j.redox.2026.104322)
Supplement: Supplementary file 1 [file mmc1.pdf]

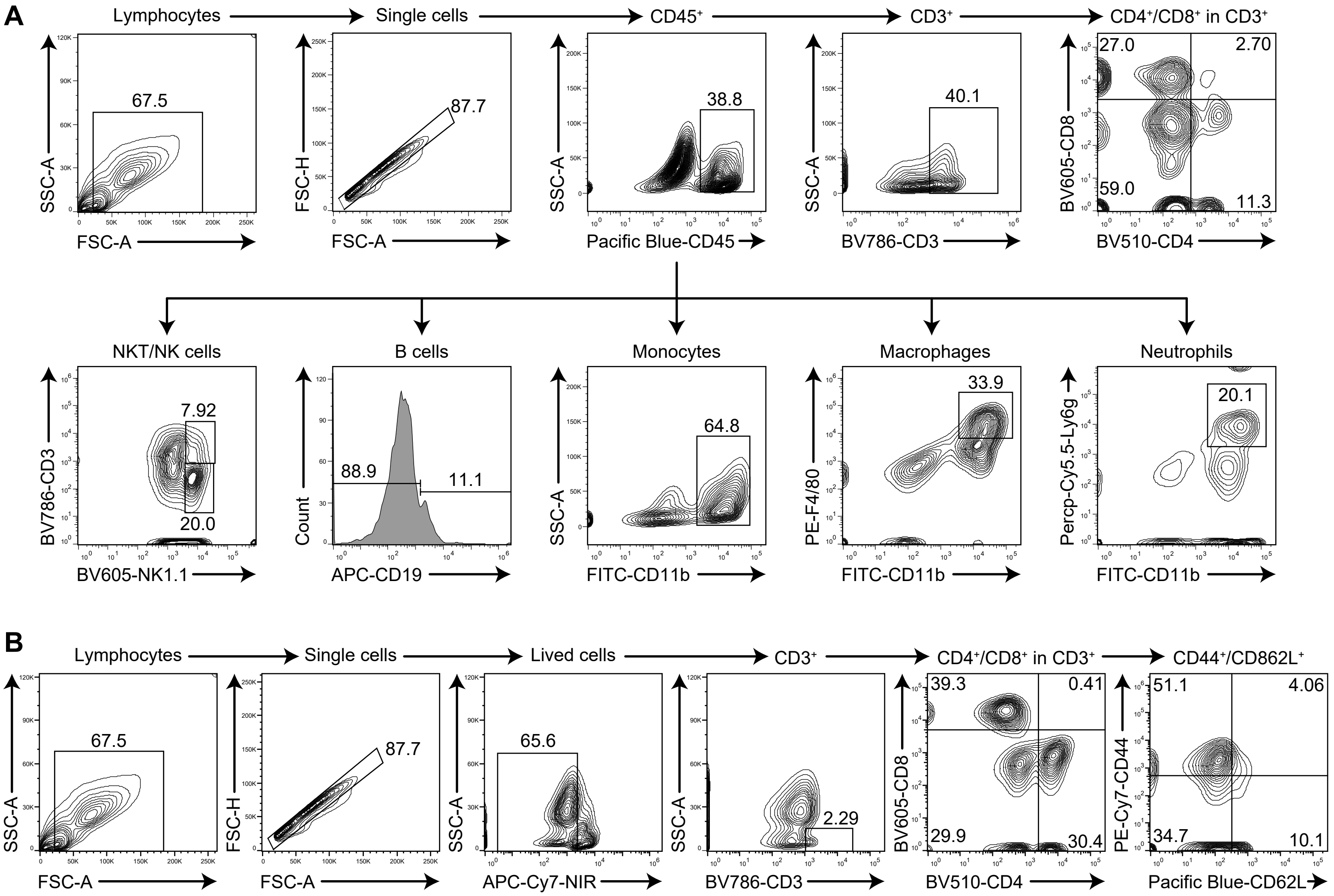

A
Lymphocytes
Single cells
CD45⁺
CD3⁺
CD4⁺/CD8⁺ in CD3⁺
SSC-A
FSC-A
67.5
FSC-H
87.7
Pacific Blue-CD45
38.8
BV786-CD3
40.1
BV605-CD8
BV510-CD4
27.0
2.70
59.0
11.3
NKT/NK cells
B cells
Monocytes
Macrophages
Neutrophils
BV786-CD3
BV605-NK1.1
7.92
20.0
Count
APC-CD19
88.9
11.1
FITC-CD11b
64.8
PE-F4/80
33.9
Percp-Cy5.5-Ly6g
20.1
B
Lymphocytes
Single cells
Lived cells
CD3⁺
CD4⁺/CD8⁺ in CD3⁺
CD44⁺/CD862L⁺
67.5
87.7
APC-Cy7-NIR
65.6
2.29
39.3
0.41
29.9
30.4
PE-Cy7-CD44
Pacific Blue-CD62L
51.1
4.06
34.7
10.1
