# Supplementary material for: Deoxycholic acid impairs mitochondrial fitness and suppresses mitophagy through Parkin ubiquitination driving CD8+ T cell Exhaustion in Colorectal Cancer
Source: Redox Biol. 2026 Jul 25;96:104322. doi: 10.1016/j.redox.2026.104322 (PMC13450440; doi:10.1016/j.redox.2026.104322)
Supplement: Supplementary file 2 [file mmc2.pdf]

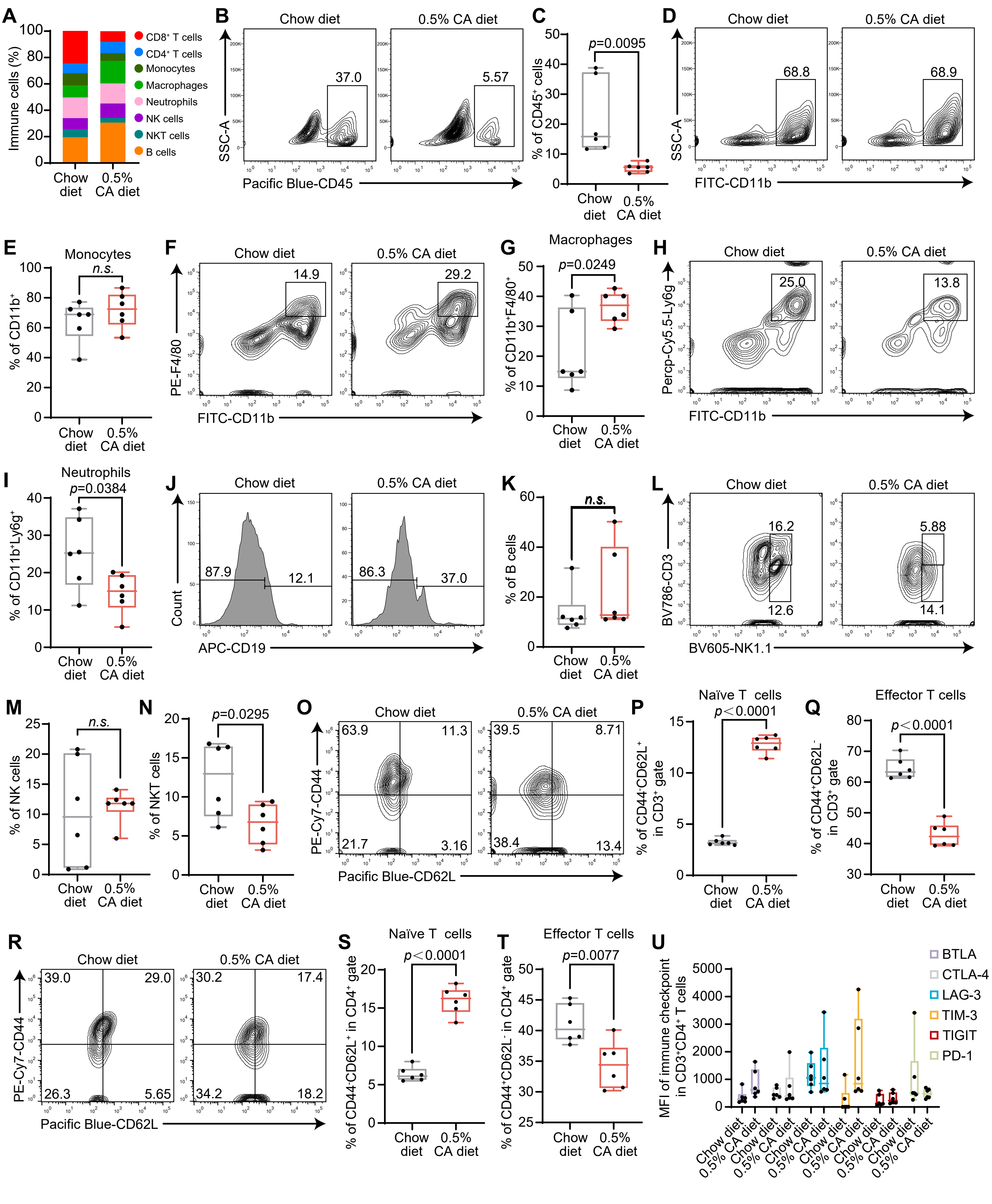

A
Immune cells (%)
CD8+ T cells
CD4+ T cells
Monocytes
Macrophages
Neutrophils
NK cells
NKT cells
B cells
Chow diet
0.5% CA diet
B
Chow diet
0.5% CA diet
SSC-A
Pacific Blue-CD45
37.0
5.57
C
p=0.0095
% of CD45+ cells
D
FITC-CD11b
68.8
68.9
E
Monocytes
n.s.
% of CD11b+
F
PE-F4/80
14.9
29.2
G
Macrophages
p=0.0249
% of CD11b+F4/80+
H
Percp-Cy5.5-Ly6g
25.0
13.8
I
Neutrophils
p=0.0384
% of CD11b+Ly6g+
J
Count
APC-CD19
87.9
12.1
86.3
37.0
K
n.s.
% of B cells
L
BV786-CD3
BV605-NK1.1
16.2
12.6
5.88
14.1
M
n.s.
% of NK cells
N
p=0.0295
% of NKT cells
O
PE-Cy7-CD44
Pacific Blue-CD62L
63.9
11.3
21.7
3.16
39.5
8.71
38.4
13.4
P
Naïve T cells
p<0.0001
% of CD44-CD62L+ in CD3+ gate
Q
Effector T cells
p<0.0001
% of CD44+CD62L- in CD3+ gate
R
39.0
29.0
26.3
5.65
30.2
17.4
34.2
18.2
S
Naïve T cells
p<0.0001
% of CD44-CD62L+ in CD4+ gate
T
Effector T cells
p=0.0077
% of CD44+CD62L- in CD4+ gate
U
MFI of immune checkpoint in CD3+CD4+ T cells
BTLA
CTLA-4
LAG-3
TIM-3
TIGIT
PD-1
