# Supplementary material for: Deoxycholic acid impairs mitochondrial fitness and suppresses mitophagy through Parkin ubiquitination driving CD8+ T cell Exhaustion in Colorectal Cancer
Source: Redox Biol. 2026 Jul 25;96:104322. doi: 10.1016/j.redox.2026.104322 (PMC13450440; doi:10.1016/j.redox.2026.104322)
Supplement: Supplementary file 3 [file mmc3.pdf]

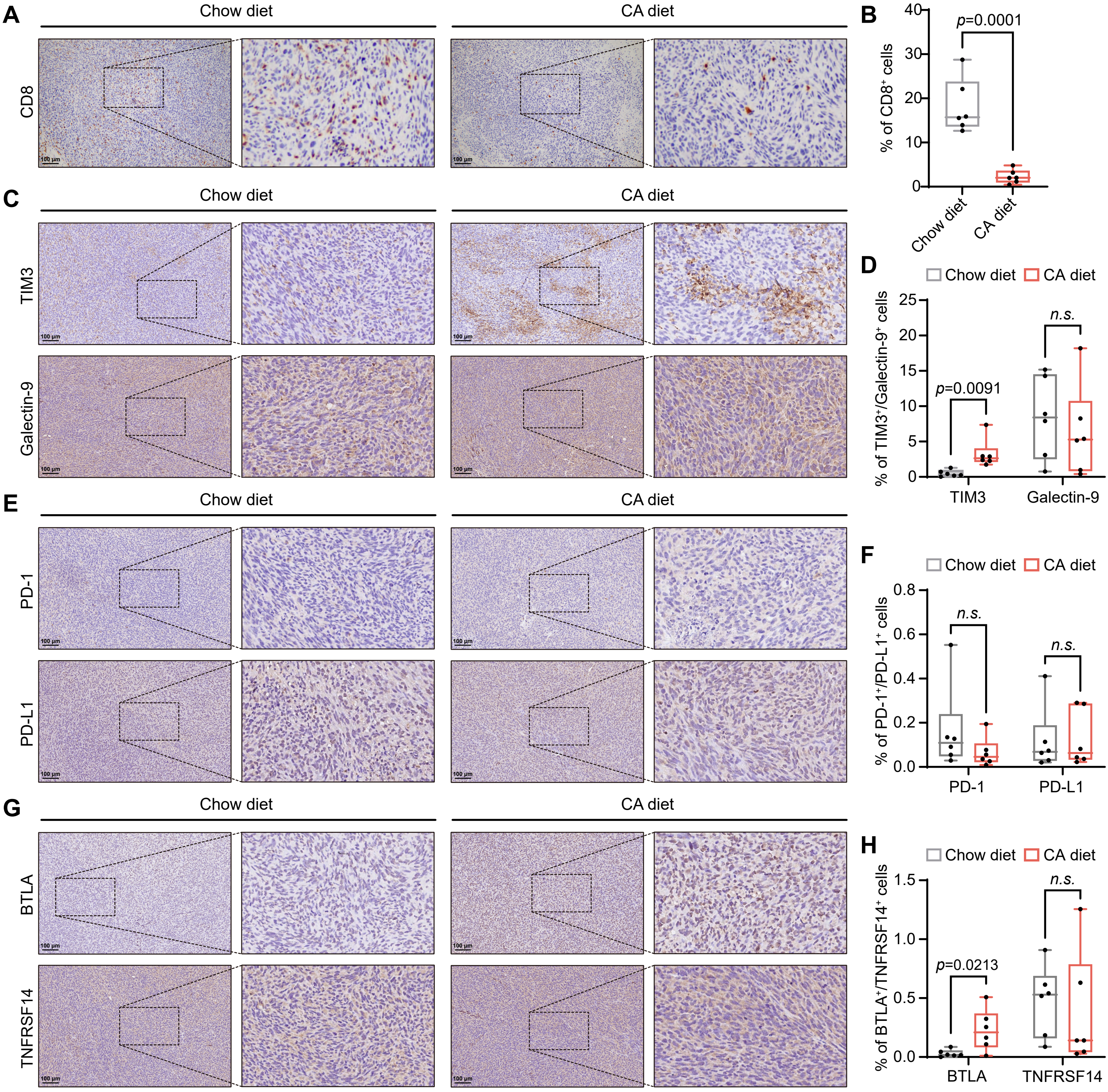
A
Chow diet
CA diet
CD8
100 μm
B
p=0.0001
% of CD8+ cells
40
30
20
10
0
Chow diet
CA diet
C
Chow diet
CA diet
TIM3
Galectin-9
D
Chow diet
CA diet
% of TIM3+/Galectin-9+ cells
25
20
15
10
5
0
p=0.0091
n.s.
TIM3
Galectin-9
E
Chow diet
CA diet
PD-1
PD-L1
F
Chow diet
CA diet
% of PD-1+/PD-L1+ cells
0.8
0.6
0.4
0.2
0.0
n.s.
n.s.
PD-1
PD-L1
G
Chow diet
CA diet
BTLA
TNFRSF14
H
Chow diet
CA diet
% of BTLA+/TNFRSF14+ cells
1.5
1.0
0.5
0.0
p=0.0213
n.s.
BTLA
TNFRSF14
