# Supplementary material for: Deoxycholic acid impairs mitochondrial fitness and suppresses mitophagy through Parkin ubiquitination driving CD8+ T cell Exhaustion in Colorectal Cancer
Source: Redox Biol. 2026 Jul 25;96:104322. doi: 10.1016/j.redox.2026.104322 (PMC13450440; doi:10.1016/j.redox.2026.104322)
Supplement: Supplementary file 4 [file mmc4.pdf]

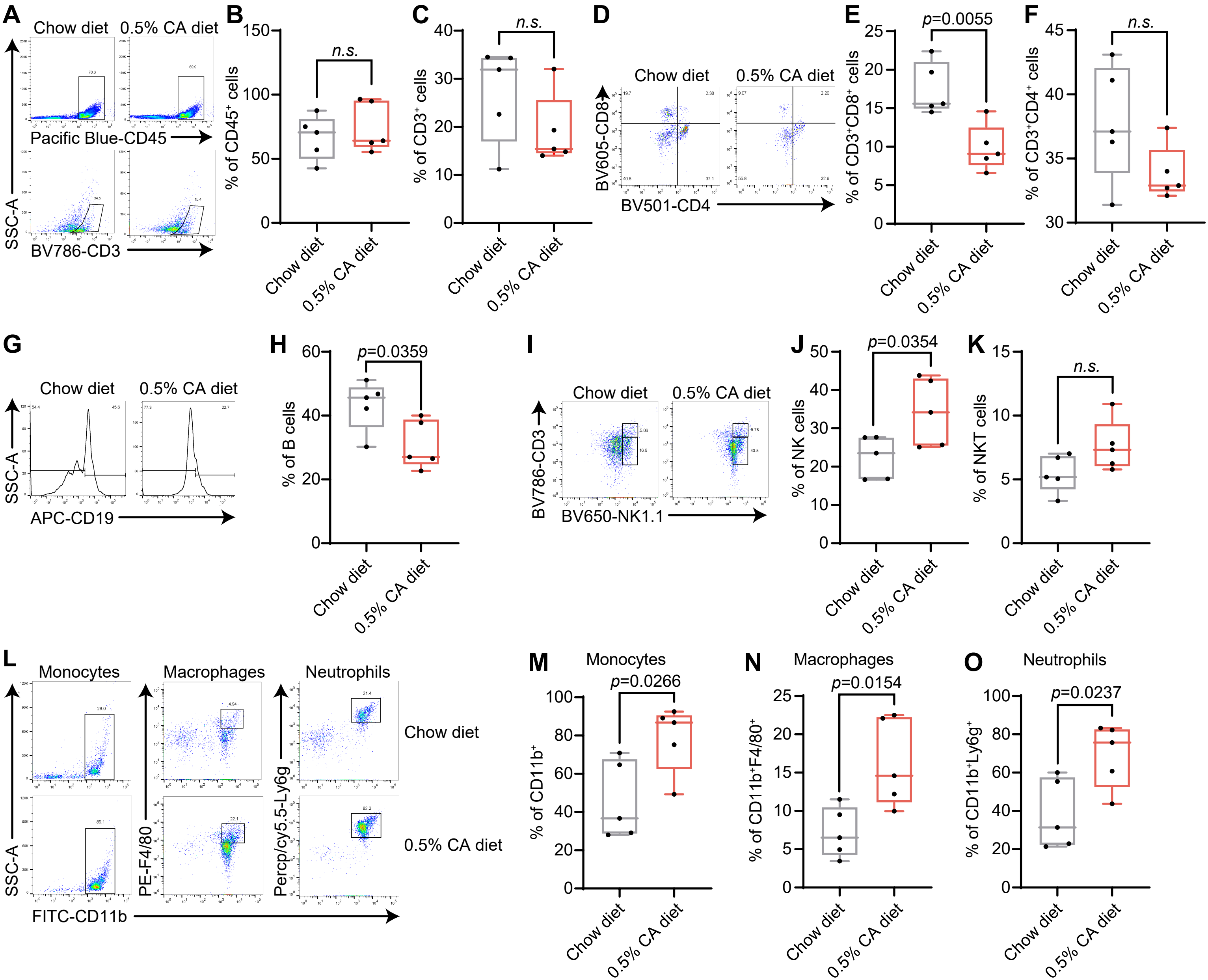

A
Chow diet
0.5% CA diet
Pacific Blue-CD45
SSC-A
BV786-CD3
B
% of CD45+ cells
n.s.
C
% of CD3+ cells
n.s.
D
Chow diet
0.5% CA diet
BV605-CD8
BV501-CD4
E
% of CD3+CD8+ cells
p=0.0055
F
% of CD3+CD4+ cells
n.s.
G
Chow diet
0.5% CA diet
SSC-A
APC-CD19
H
% of B cells
p=0.0359
I
Chow diet
0.5% CA diet
BV786-CD3
BV650-NK1.1
J
% of NK cells
p=0.0354
K
% of NKT cells
n.s.
L
Monocytes
Macrophages
Neutrophils
Chow diet
0.5% CA diet
SSC-A
PE-F4/80
Percp/cy5.5-Ly6g
FITC-CD11b
M
Monocytes
% of CD11b+
p=0.0266
N
Macrophages
% of CD11b+F4/80+
p=0.0154
O
Neutrophils
% of CD11b+Ly6g+
p=0.0237
Chow diet
0.5% CA diet
