# Supplementary material for: Deoxycholic acid impairs mitochondrial fitness and suppresses mitophagy through Parkin ubiquitination driving CD8+ T cell Exhaustion in Colorectal Cancer
Source: Redox Biol. 2026 Jul 25;96:104322. doi: 10.1016/j.redox.2026.104322 (PMC13450440; doi:10.1016/j.redox.2026.104322)
Supplement: Supplementary file 5 [file mmc5.pdf]

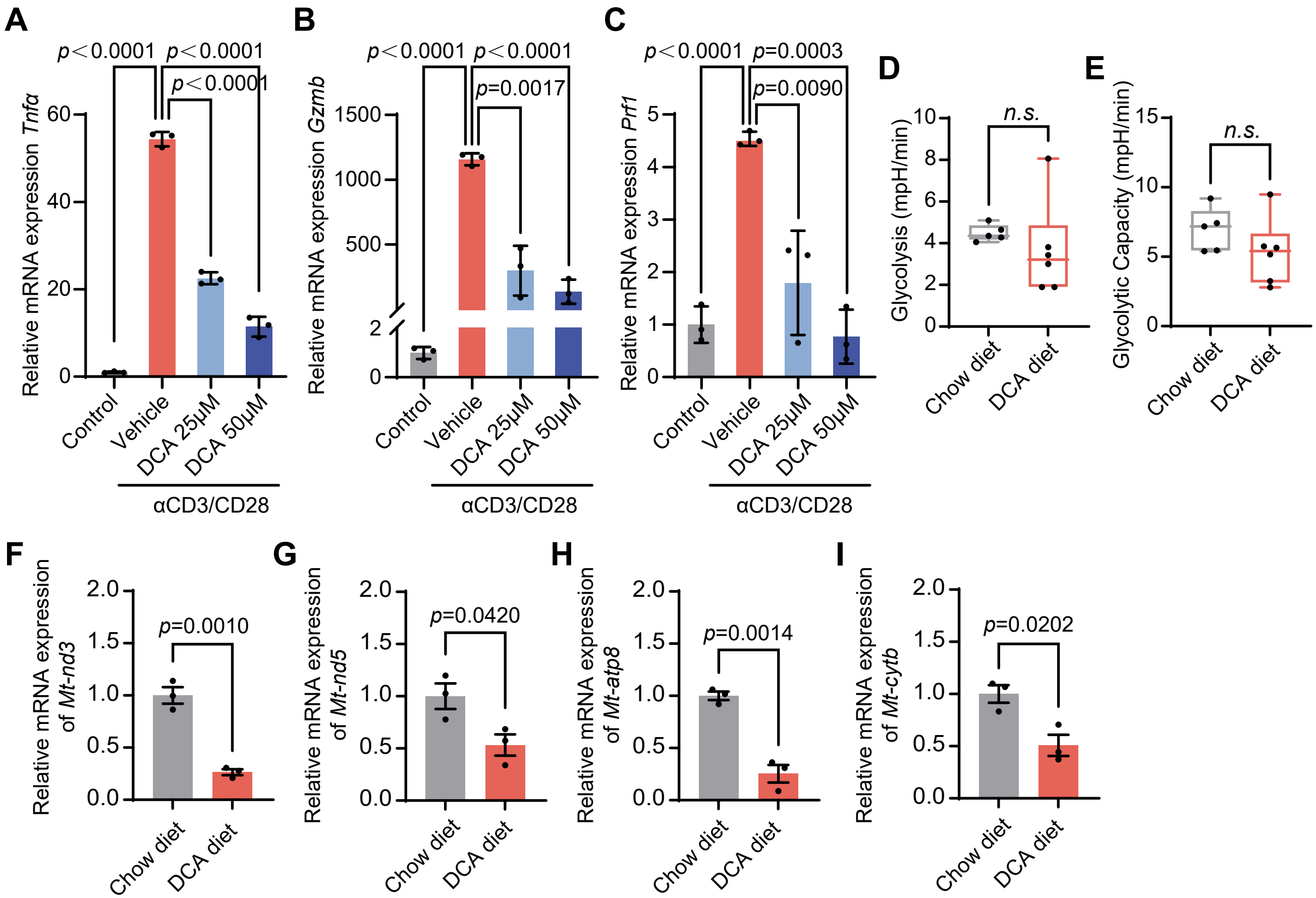

A
B
C
D
E
F
G
H
I
Relative mRNA expression Tnfα
Relative mRNA expression Gzmb
Relative mRNA expression Prf1
p<0.0001
p<0.0001
p=0.0017
p=0.0003
p=0.0090
Control
Vehicle
DCA 25μM
DCA 50μM
αCD3/CD28
Glycolysis (mpH/min)
Glycolytic Capacity (mpH/min)
n.s.
Chow diet
DCA diet
Relative mRNA expression of Mt-nd3
Relative mRNA expression of Mt-nd5
Relative mRNA expression of Mt-atp8
Relative mRNA expression of Mt-cytb
p=0.0010
p=0.0420
p=0.0014
p=0.0202
