# Supplementary material for: Deoxycholic acid impairs mitochondrial fitness and suppresses mitophagy through Parkin ubiquitination driving CD8+ T cell Exhaustion in Colorectal Cancer
Source: Redox Biol. 2026 Jul 25;96:104322. doi: 10.1016/j.redox.2026.104322 (PMC13450440; doi:10.1016/j.redox.2026.104322)
Supplement: Supplementary file 6 [file mmc6.pdf]

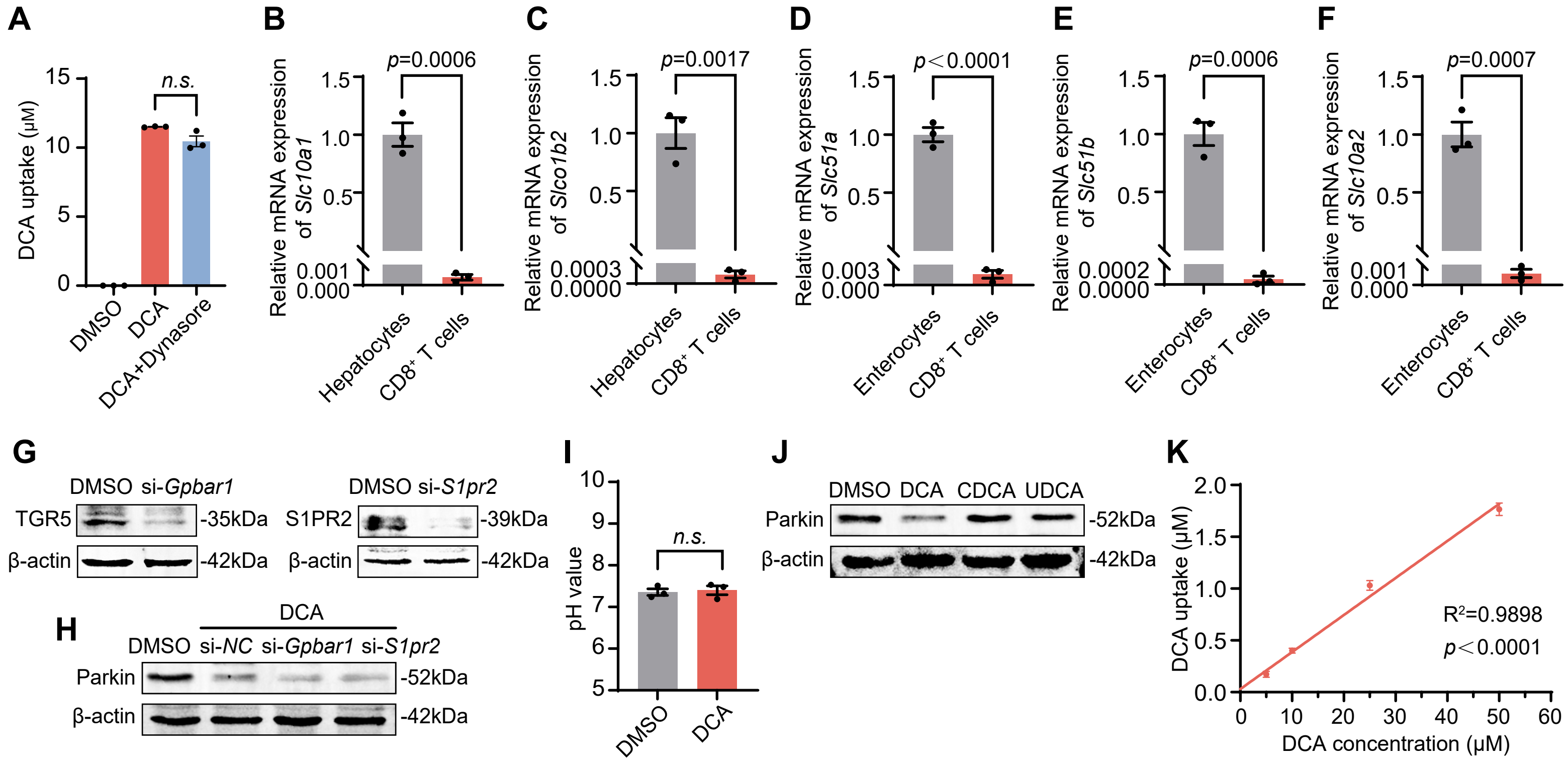

A
DCA uptake (μM)
15
10
5
0
n.s.
DMSO
DCA
DCA+Dynasore
B
Relative mRNA expression of Slc10a1
p=0.0006
Hepatocytes
CD8+ T cells
C
Relative mRNA expression of Slco1b2
p=0.0017
Hepatocytes
CD8+ T cells
D
Relative mRNA expression of Slc51a
p<0.0001
Enterocytes
CD8+ T cells
E
Relative mRNA expression of Slc51b
p=0.0006
Enterocytes
CD8+ T cells
F
Relative mRNA expression of Slc10a2
p=0.0007
Enterocytes
CD8+ T cells
G
DMSO si-Gpbar1
TGR5 -35kDa
β-actin -42kDa
DMSO si-S1pr2
S1PR2 -39kDa
β-actin -42kDa
H
DCA
DMSO si-NC si-Gpbar1 si-S1pr2
Parkin -52kDa
β-actin -42kDa
I
pH value
n.s.
DMSO
DCA
J
DMSO DCA CDCA UDCA
Parkin -52kDa
β-actin -42kDa
K
DCA uptake (μM)
R²=0.9898
p<0.0001
DCA concentration (μM)
